# Cathepsin L promotes secretory IgA response by participating in antigen presentation pathways during *Mycoplasma Hyopneumoniae* infection

**DOI:** 10.1371/journal.pone.0215408

**Published:** 2019-04-15

**Authors:** Ning Zhang, Peng Gao, Bao Yin, Jiahe Li, Tong Wu, Yu Kuang, Wenxue Wu, Jinxiang Li

**Affiliations:** 1 College of Veterinary Medicine, China Agricultural University, Beijing, China; 2 Department of Critical Care Research, University of Texas MD Anderson Cancer Center, Texas, TX, United States of America; 3 Department of Veterinary Public Health Safety and Management, Chinese Academy of Agricultural Sciences, Beijing, China; Instituto Butantan, BRAZIL

## Abstract

Cathepsin L (CTSL) has been proved to help contain leishmaniasis and mycoplasma infection in mice by supporting cellular immune responses, but the regulatory functions of CTSL on mucosal immune responses haven’t been tested and remain undefined. Here, we investigated the effects of CTSL on SIgA responses and invariant chain (Ii) degradations in the co-cultured swine dendritic cells (DCs) and B cells system *in vitro*. When the cells system were transfected with vector CTSL-GFP or incubated with recombinant CTSL (rCTSL) before they were infected with *Mycoplasma hyopneumoniae* (*M*.*hp*), SIgA significantly increased and Ii chain was degraded into smaller intermediates, while SIgA decreased when CTSL was knockdown or inhibited with E64. To confirm the SIgA responses promoted by CTSL contribute to the resistance to mycoplasma pneumonia, pigs injected with rCTSL before they were challenged with *M*.*hp*, showed milder clinical symptoms and histopathological damage of lungs, less mycoplasma burden together with higher secretion of SIgA, percentages of CD4^+^ T cells and level of MHC II molecules comparing with the group without rCTSL. Collectively, these results suggested that rCTSL could provide effective protection for piglets against mycoplasma pneumonia by enhancing *M*.*hp*-specific mucosal immune responses through its role in antigen presentation by processing the invariant chain.

## Introduction

Mucosal surfaces of the respiratory tract and gastrointestinal tract are the first line of defense to prevent the infection of pathogens and clear the evaded microorganism by excreting active components including secretory immunoglobulin A (SIgA) [1, 2]. Experimental evidence showed that SIgA response provides effective protection especially for the cases of chronic mucosal infections and reinfections of many bacteria, virus, parasites and fungi [3–6]. Mycoplasma, unlike virus or bacteria, leads infection by firmly adhering to respiratory epithelial cells. To clear mycoplasma, mucosal immunity instead of systemic immunity plays a key role during infection [7]. And SIgA is the first sign of mycoplasma infection which raises the strategy considerations about the induction of SIgA by vaccination [8, 9].

As a key link in the process of inducing immune response, major histocompatibility complex class II (MHC II) molecules function to present antigen to CD4^+^ T lymphocytes generating helper T cell responses, which activate unprimed B cells to secrete IgG and IgA [10]. MHC II molecules are assembled in the endoplasmic reticulum with the assistance of the chaperone molecule invariant chain (Ii), and transported to an endocytic compartment where Ii is cleaved sequentially to discrete intermediates such as p22, p10 and class II-associated leupeptin-induced peptide (CLIP) fragments [11, 12]. CLIP remains bound to the MHC class II peptide binding groove until it is exchanged for resident antigenic peptides [13, 14]. Therefore, endosomal MHC class II-Ii complexes can only become competent for antigenic peptide loading after Ii chain processing.

A number of cysteine cathepsins have been proven to play crucial roles in the pathway of Ii processing by studies with protease inhibitors and knockout mice [15–17]. Cathepsin L (CTSL) is involved in the late stage of Ii degradation, since Ii intermediates, p12 or small leupeptin-induced peptide (SLIP) accumulated due to the deficiency of CTSL [16–19]. CTSL has also been implicated in regulatory events relating to immunological responses [18, 20, 21], infectious diseases [22–24], cancer [25, 26], diabetes [27] and other pathological processes [28–30]. Recent studies revealed that CLIK148, a specific inhibitor of CTSL, enhances the development of Th2-type immune responses and leads to exacerbation of leishmaniasis in mice, and CTSL helps contain mycoplasma infection by supporting lymphangiogenesis and cellular immune responses instead of humoral immune responses [28]. However, it still remains obscure whether CTSL contributes to the protections from the infection by mucosal immune responses or not since there are little direct or indirect evidences available for now.

Taking into account that CTSL is involved in MHC II–dependent immune responses, we hypothesized that CTSL may provide protection against respiratory tract infections of bacterial pathogens by improving mucosal immunity. To test these predictions, we investigated the preventive effects of recombinant CTSL (rCTSL) on mycoplasma pneumonia of swine which is the most approximate model to humans [31, 32], and the effects of rCTSL and native CTSL on SIgA responses and Ii degradations in the co-cultured dendritic cells (DCs) and B cells model *in vitro*. These results revealed that rCTSL enhanced the resistance to mycoplasma pneumonia for swine, and CTSL promoted SIgA responses and Ii degradation significantly.

## Materials and methods

### Animals and welfare

Thirty days old crossbred pigs obtained from Swine Breeding Center in Beijing were housed in groups in a confined university facility under ethologically and hygienically ideal conditions and acclimatized for two weeks. Animal use and animal trials in this study were approved by Beijing Municipal Committee of Animal Management and Ethics Committee of China Agricultural University (approval number: CAU20140629-2). All experiments strictly followed the recommended guidelines by the Ethics Committee of China Agricultural University. Animal cadavers were disposed of in compliance with the Rules for Working in Experimental Animal Facilities and Valid Waste Regulations. Pigs were anaesthetized with atropine (0.05 mg/kg), ketamine (5 mg/kg) and propofol (3 mg/kg), which were provided by Veterinary Hospital of China Agricultural University before experiments. All efforts were used to reduce the pain and adverse effect of the animals.

### Preparation and enzyme activity of recombinant protein dx.doi.org/10.17504/protocols.io.zf7f3rn

The coding sequence of CTSL was amplified by PCR with the primer pairs (CTSL-R:*5’-CGCGTCGACATGGCTCCAAAACTTGATC-3*; CTSL-F:*5’-CGCGCGGCCGCTCACACGGTGGGATAGCTGGC-3*) designed according to the sequence (NC_010452.3) as described in S1 Methods. CTSL sequence was ligated with pET28a vector (Invitrogen, USA) and purified with Ni-NTA agarose (QIAGEN, Valencia, USA) after expressed. The enzymatic activity of rCTSL was measured with the substrate benzyloxycarbonyl-phenylalanyl-arginine 4-methyl-7-coumarylamide (Z-Phe-Arg-MCA) (Enzo Life Sciences, Switzerland). To make recombinant protein activated, 100 ng aliquot of rCTSL was incubated in 150 μl buffer solution (10 mM Tris-HCl and 0.1M NaH_2_PO_4_, pH 5.5) at 37°C for 1 h. Freshly prepared substrates (50 μl) were added to a final concentration of 10 mM. The fluorescence signal liberated by hydrolysis was detected using a spectrofluorometer. The enzymatic activity was expressed as the increased fluorescence units/min during incubation. E64 (Sigma-Aldrich, St. Louis, MO, USA) a cysteine proteinase inhibitor, was used as a control to prove the recombinant protein is a cysteine proteinase.

### Western blots

Recombinant CTSL protein was quantified using Pierce BCA Protein Assay Kit (Takara) and the 20–30 μg sample per lane was subjected to western blot analysis. Briefly, PVDF membranes were incubated for 2 h with mouse anti-Cathepsin L antibody [33/2] (1:1000 dilution in PBST) (Abcam, USA) and horseradish peroxidase (HRP)-conjugated goat anti mouse IgG (HRP) (1:2000 dilution in PBST) (Abcam, USA), detected by *EasySee* Western Blot Kit (TransGen Biotech, Beijing, China).

### Sandwich ELISA construction

Mouse anti-CTSL monoclonal antibody (mAb) and rabbit anti-CTSL polyclonal antibody (pAb) were produced by our lab at China Agricultural University. The concentrations of CTSL were analyzed with sandwich ELISA as described in S2 Methods.

### Isolation and culture of DC cells and B cells

For isolation of DCs, peripheral blood mononuclear cells (PBMC) were isolated and cultured (S3 Methods) firstly. Then, monocytes were enriched by MACS separation. CD11b+ cells were positively selected with magnetic microbeads accordingly to manufacturer’s instructions (Miltenyi Biotec, Auburn, CA), and cultured in a complete RPMI 1640 medium with 20 ng/ml of recombinant porcine granulocyte-macrophage colony-stimulating factor (rpGM-CSF, Invitrogen, CA, USA) and 10 ng/ml of recombinant porcine interleukin 4 (rpIL-4, Invitrogen, CA, USA). Half of the culture medium was replaced with fresh medium every two days. At 7 d, monocytes differentiated to immature monocyte-derived DC (Mo-DC), then become mature after stimulated with 2 μg/ml of lipopolysaccharide (LPS, Sigma) for 24 h.

For isolation of B cells, pig spleens were disaggregated in RMPI-1640 using a mortar and pestle and sifted through a cell filter, followed by depletion of RBC with ammonium chloride solution. CD19+ B cells were separated by MACS with mouse anti-pig CD19 antibody. B cells were cultured for 5 days in RPMI-1640 medium with 10% FBS and 25 μg/ml of rpIL-4, 50 μg/ml interferon γ (IFN-γ, Invitrogen, CA, USA) and 50 μg/ml CD40L (DAKEWE, China). Half of the medium was replaced by fresh complete medium every two days. At 7 d, B cells were stimulated for 24 h with 100 ng/ml CpG-ODN 2006 (Invitrogen, CA, USA).

### Flow cytometric analysis

Surface markers of DCs and B cells were analyzed by flow cytometry. Procedures of flow cytometry can be seen in online Supplementary Methods. For DCs, labeling strategies were as follows:(1) anti-CD1 antibody (FITC) [clone 76-7-4], mouse anti pig CD172a antibody [clone BL1H7]; (2) anti-CD4 antibody (FITC) [clone 74-12-4], anti-CD8 antibody (Phycoerythrin) [clone MIL-12]; (3) anti-CD11b antibody (FITC) [clone 2F4/11], anti-porcine MHC Class II DQ antibody [clone K274.3G8]; (4) anti-CD14 antibody (Phycoerythrin) [clone TÜK4], anti-CD21 antibody (Alexa Fluor647) [clone LT21]; (5) anti-CD163 antibody (Phycoerythrin) [clone 2A10/11 ]. B cells were labeled with strategy: (1) anti-CD1 antibody (FITC) [clone 76-7-4], anti-CD20 antibody [clone MEM-97]; (2) anti-CD4 antibody (FITC) [clone 74-12-4], anti-CD8 antibody (Phycoerythrin) [clone MIL-12]; (3) anti-CD11b antibody (FITC) [clone 2F4/11], anti-CD79a antibody (PerCP/Cy5.5) [clone HM47]; (4) anti-CD21 Santi-porcine MHC Class II DQ antibody [clone K274.3G8].

The presence of CD4^+^ T cells and CD8^+^ T cells in PBMC was also analyzed with mouse monoclonal anti-porcine CD4-FITC [clone 74-12-4] and mouse monoclonal anti-pig CD8- PE/Cy5 [clone 76-11-2] separately, as described in S4 Methods.

### Co-culture of DCs and B cells *in vitro*

For the co-culture of DCs and B cells, differentiated immature DC cells (iDCs) and B cells were harvested respectively at 6 d after culture respectively, and counted by staining with 0.4% Trypan blue (STEMCELL, Canada). About 1.0×10^4^ iDCs and 1.0×10^5^ B cells were seeded in the same wells of 96-well culture plates, and RPMI-1640 complete medium with 20 ng/ml rpGM-CSF, 25 μg/ml of rpIL-4, 50 μg/ml IFN-γ and 50 μg/ml CD40L were added. After co-cultured for 48 h, cells were under different treatment. In group 1, cells were infected with *M*.*hp* for 2 h. In group 2, cells were treated with rCTSL protein for 1 h followed by infection with *M*.*hp* for 2 h. In group 3, cells were transfected with eukaryotic plasmid CTSL-GFP for 48 h followed by infection with *M*.*hp* for 2 h. In group 4, cells were treated with E64 for 1 h followed by infection with *M*.*hp* for 2 h. In group 5, cells were transfected with px458-cas9-CTSL vector for 48 h followed by infection with *M*.*hp* for 2 h. At the same time, B cells and DCs were cultured separately as controls. The cell supernatants were collected for the detections of various cytokines and SIgA, and the pellets were collected for the detections of CTSL and Ii chain.

### Cells transfection

The eukaryotic expression vector CTSL-GFP was constructed as described in S5 Methods and used for transfection. Knockdown vector was conducted with CRISPR-Cas9 plasmid. According to the gene of porcine CTSL (Gene ID: 396926, S1 Methods), sgRNAs were designed by Biomics Biotechnologies Company. We choose *9r-AGGGCGGTCAGGAAGAGTGA*; and *253r-CACCTGCCTGAATTCTTCATTGG* as double targets and constructed the plasmid px458-253r used for the knockdown CTSL by the methods as previously described [33, 34]. Cells transfection was performed as described in manufacturer's instructions. Briefly, cells were transfected with 10 μl of Lipofectamine 2000 in 240 μl RMPI 1640 (GIBCO) containing conditional vectors. Empty vector was used as a transfection control.

### Cytokines detection

Cytokines related to the immunological response were measured in cell culture supernatants using Pig Transforming Growth Factor β1 (TGF-β1) (CSB-E06843p), Pig Interferon IFN-γ (CSB-E06794p), Pig Interleukin 6 (CSB-E06786p) and Pig interleukin 10 (CSB-E06779p) ELISA kits purchased from CUSABIO (Wuhan, China) according to manufacturer’s instructions.

### Immunoprecipitation assay dx.doi.org/10.17504/protocols.io.zgbf3sn

Cells were harvested and lysed in NP-40 lysis buffer containing 0.5% NP-40, 150 mM NaCl, 50 mM Tris-HCL pH 8.0 for 1h on ice, and centrifuged at 12,000 rpm for 20 min. The cell supernatants were then precleared with protein A/G sepharose beads and immunoprecipitated with anti-porcine MHC class II antibody [K274.3G8] and protein A/G sepharose beads overnight at 4°C with gentle rotation. The beads were washed five times with NP-40 lysis buffer and the proteins bounded to the beads were separated by 10–20% tricine gel and analyzed by silver stain.

### Animal experiment

Eighteen piglets confirmed to be *M*.*hp* free by antibody detection were randomly divided into three groups. Control group was six pigs injected intratracheally with 3 ml of phosphate buffered saline (PBS); *M*.*hp* group was six pigs challenged with 3ml of PBS containing 5×10^7^ CCU of *M*.*hp* strain; rCTSL+*M*.*hp* group was six pigs which first injected via bronchofiberscopy with 20 μg of rCTSL protein, and then challenged with 3ml of PBS containing 5×10^7^ CCU of *M*.*hp* strain. The three groups of animals were housed separately. Rectal temperatures and clinical symptoms of each piglet were monitored and scored as previously described [35]. Gross lung lesions were estimated immediately when the pigs were autopsied at 21 day post-infection (DPI). The scoring system for lung gross lesion was employed as previously described [36–38]. To detect the burden of live mycoplasma, lung homogenates were serially diluted with mycoplasma medium and CCU were recorded between 7 to 10 d after culture. Bronchoalveolar lavage fluids (BALFs) were performed as previously described [39, 40] with an electronic fiberoptic bronchoscope (OLYMPUS, X2-5) inserted in the lung lobe.

### Microscopic lesion and immunohistochemistry (IHC) examination dx.doi.org/10.17504/protocols.io.zgwf3xe

Tissues were collected at necropsy, fixed with 4% paraformaldehyde solution at room temperature for 48 h and then processed by routine histopathological procedures. Each sample was examined on three sections. One section was stained with hematoxylin and eosin (H&E) for observing pathological changes, and the H&E stained sections were blindly evaluated by a veterinary pathologist and the distribution and severity of interstitial pneumonia were recorded as previously described [36, 41].

The other two sections were subjected to detect CTSL and MHC class II respectively by IHC staining with mouse anti-Cathepsin L antibody [33/2] (1:50 dilution) and anti-porcine MHC class II antibody [K274.3G8] (1:100 dilution) respectively. The number of positive cells in section was executed as previously described [35, 42].

### Mycoplasma-specific immunoglobulin detection

Mycoplasma-specific IgG, IgM and SIgA levels in nasal, serum and BALF samples were detected by pig IgG ELISA Kit (CSB-E06804p), IgM ELISA Kit (CSB-E06805p) and SIgA ELISA Kit (CSB-E12063p) purchased from CUSABIO BIOTECH CO., LTD. (Wuhan, China).

### Statistical analysis

The significant differences of the in vivo experiment and animal trials were analyzed using t-test, two-way ANOVA in the GraphPad Prism (version 5.0) software. Differences were considered statistically significant at a value of P < 0.05 and extremely significant at a value of *P*<0.01 or *P*<0.001.

## Results

### The harvest and phenotype of DCs and B cells *in vitro*

**Fig 1:**
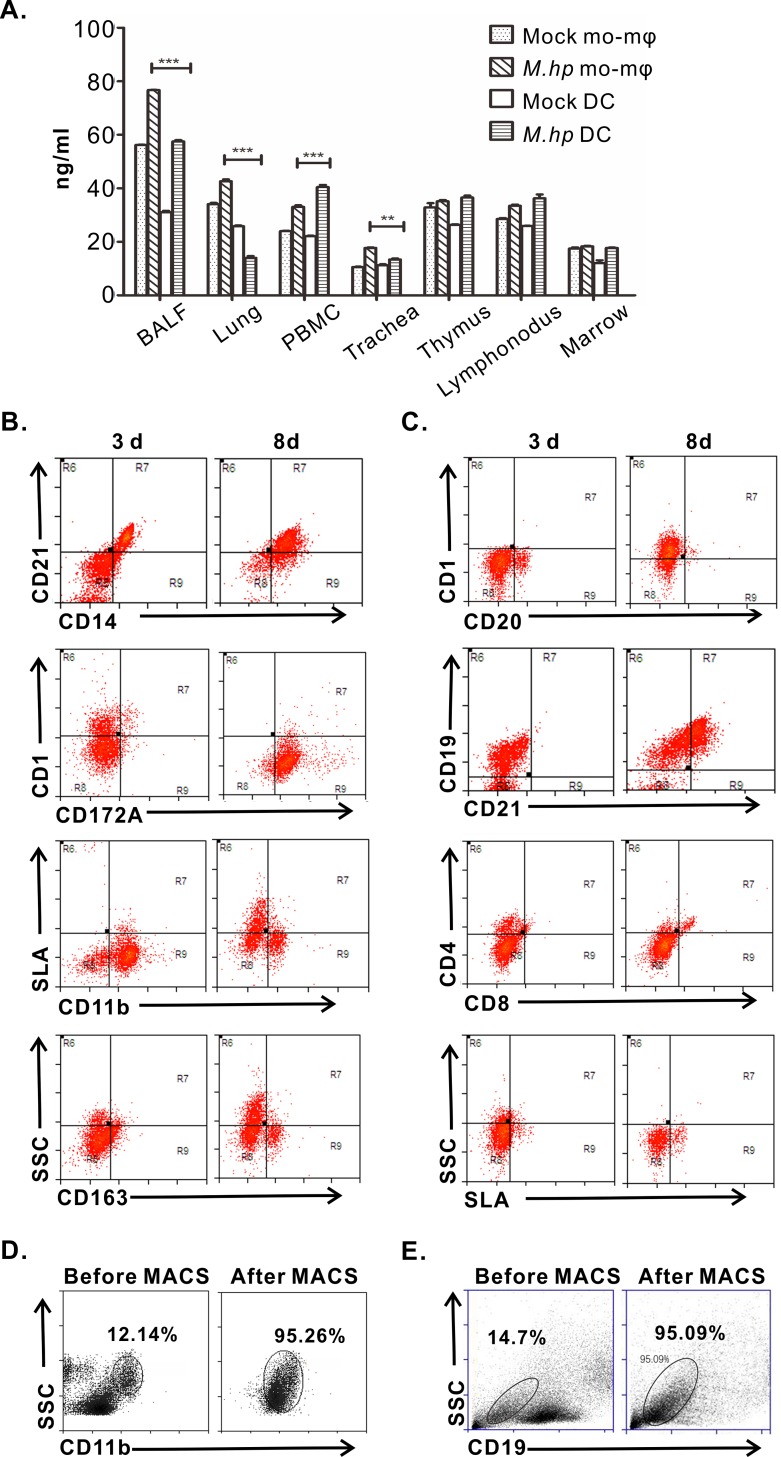
Isolation and classification of DCs and B cells *in vitro*. **(A)** CTSL was measured in different tissue cells by sandwich ELISA. Values represented as mean ± SEM from three independent experiments (**p<0.01, ***p<0.001). **(B)** Phenotype of DCs: on day 3, phenotype was CD11b^+^ CD14^+^ CD4^+^ CD8^-^ SLA-DR^low^ and CD11b^+^ CD21^+^ CD8^+^ CD4^low^SLA-DR^high^ on day 8. **(C)** Phenotype of B cells: CD19^+^ CD20^+^ CD4^low^ on day 3 and CD19^+^ CD20^+^ CD21^+^ CD8^low^ SLA-DR^low^ on day 8. **(D)** Percentage of Mo-DC cells: before MACS, there was 12.14% Mo cells in PBMC; after MACS, the purity was 95.26%. **(E)** Percentage of B cells: B cells was 14.7% before MACS; after MACS, the purity was 95.09%. Data are representative of three independent experiments.

To investigate the exact roles of CTSL on the mucosal immune response to *M*.*hp* infection, we sought to develop a co-culture model of antigen presentation cells (APCs) and B cells. First, the concentrations of CTSL in DCs and macrophages in various tissues of the pigs were detected by sandwich ELISA in order to select the ideal type of APCs. There were greater differences on the levels of CTSL in DCs in most tissues between the infection group and control group, indicating stronger responses of CTSL were present in DCs rather than macrophages (Fig 1A). When cultured *in vitro*, the morphology of Mo-DCs was small, disaggregated and rounded at 1 d with clusters formed at 3 d, and partial cells grew in suspension with obvious dendritic protrusions since day 5 (S1 Fig). The surface markers of DCs were CD11b^+^ CD14^+^ CD21^+^ CD1^+^ CD163^+^ for immature cells at 3 d and CD11b^+^ SLA-DR^+^ CD21^+^ CD14^+^ CD172a^+^ for mature cells at 8 d after stimulated with LPS (Fig 1B). B cells were small and round (S2 Fig) and the phenotype was CD19^+^ CD20^+^ CD4^low^SLA-DR^low^ at 3 d and CD19^+^ CD20^+^ CD21^+^ SLA-DR^+^ at 8 d after stimulated with CpG-ODN (Fig 1C). Before co-culture, immature Mo-DCs and B cells were harvested by MACS and purity were 95.26% (Fig 1D) and 95.09% (Fig 1E) respectively.

### CTSL promotes SIgA response to *M*.*hp* infection in DCs+B cells

**Fig 2:**
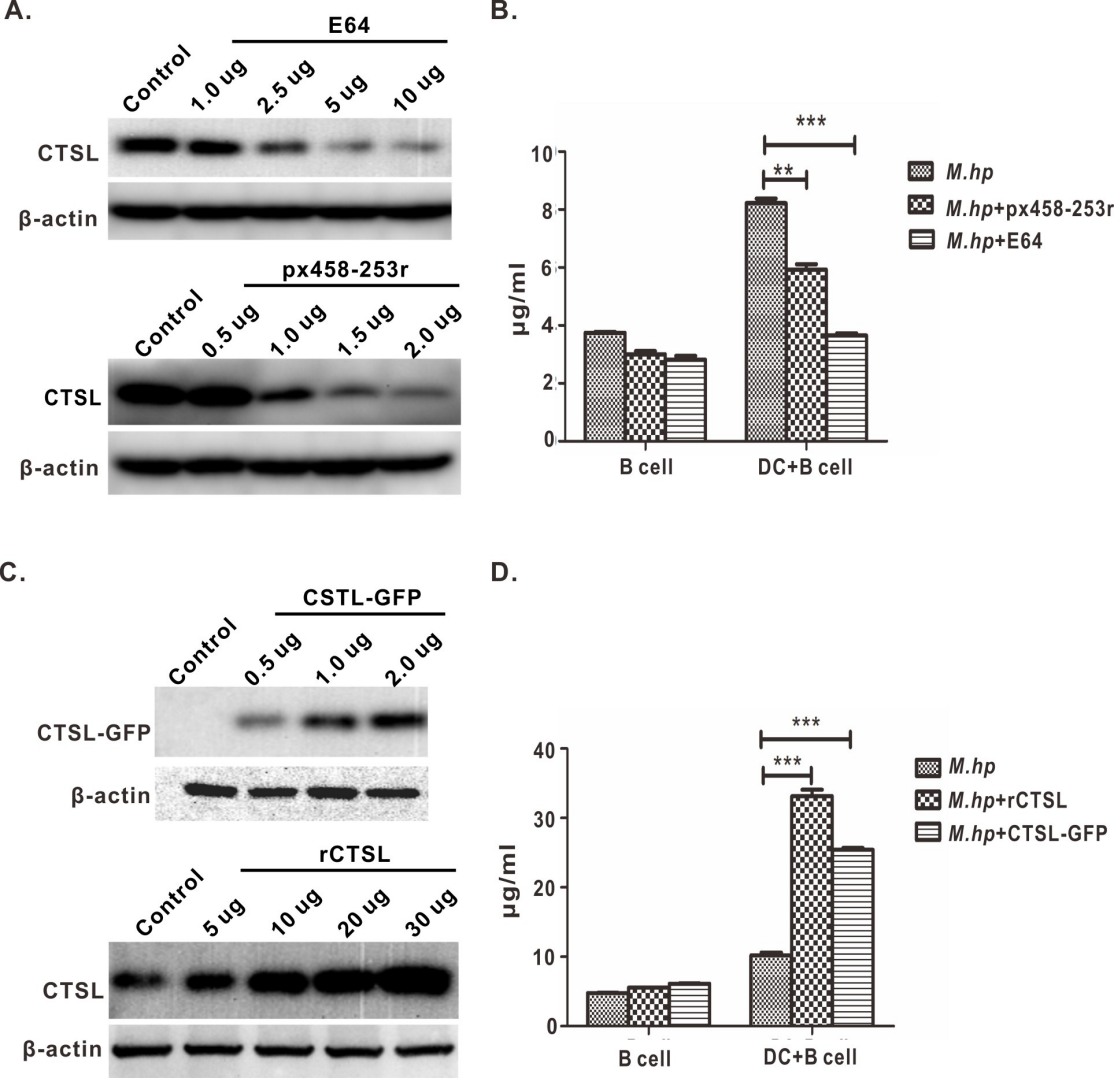
Effect of CTSL on SIgA secretion. **(A)** Knockdown or inhibit CTSL: CTSL was partially or almost completely knockdown when 1.5 μg or 2 μg plasmid px458-253r were added in a well of a 24 well plates; 5 μg or 10 μg E64 could inhibit CTSL. **(B)** Levels of SIgA significantly decreased when DCs+B cells were incubated with 2 μg plasmid px458-253r or 10 μg E64. **(C)** Overexpression or injection with rCTSL: CTSL concentration increased when injected with 30 μg rCTSL, while only slightly increased with 5 μg rCTSL; CTSL was overexpressed when transinfected with eukaryotic plasmid CTSL-GFP from 0.5 μg to 2.0 μg. **(D)** Levels of SIgA increased significantly when DCs+B cells were treated with 30 μg rCTSL or 2.0 μg CTSL-GFP. Data represent means ± SEM from three independent experiments (**p<0.01, ***p<0.001).

Mo-DCs and B cells were co-cultured *in vitro* and infected with *M*.*hp*. In the co-cultured cells, the levels of CTSL conditional knockdown after CTSL transfected with 1.5 or 2 μg CRISPR/Cas9 plasmid px458-253r and dropped obviously after treated by 5 or 10 μg inhibitor E64 (Fig 2A). The concentrations of SIgA decreased about 50% and 75% accordingly since the level of CTSL decreased in co-cultured cells (Fig 2B). On the contrary, CTSL increased significantly in the DCs+B cells treated with 30 μg rCTSL or transfected with 2.0 μg CTSL-GFP (Fig 2C), and SIgA increased almost 3 fold and 2.5 fold respectively (Fig 2D). In the B cells, all the four kinds of treatments didn’t have significant impacts on the levels of SIgA (Fig 2B and 2D). The above data indicated that there is high positive correlation between the changes of CTSL and SIgA in DCs+B cells infected with *M*.*hp*, and CTSL promoted SIgA response as we supposed.

### CTSL regulated cytokines secretion in DCs+B cells

**Fig 3:**
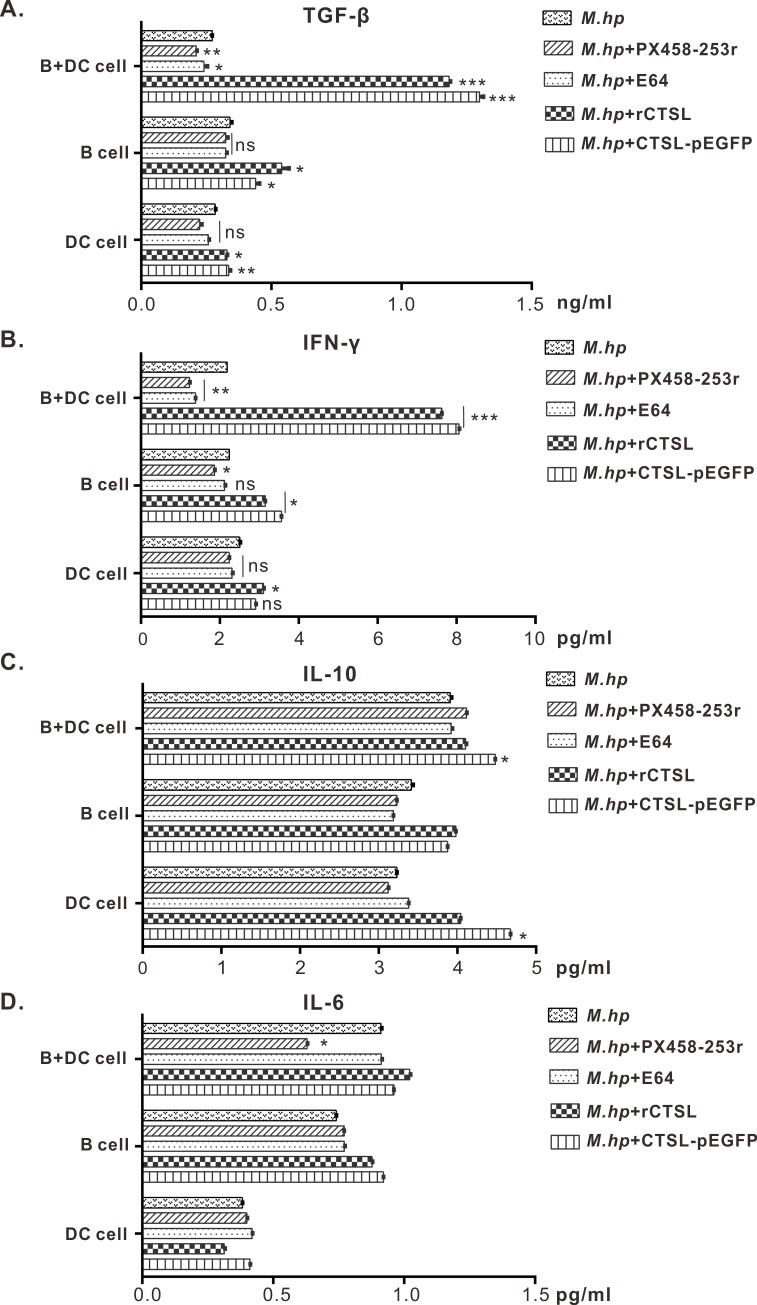
Cytokines concentrations were measured in cell culture’s supernatants by ELISA. **(A)** Concentration of TGF-β: DCs+B cells pretreated with rCTSL protein or CTSL-GFP eukaryotic plasmid had a significant increase, while the concentration decreased when treated with inhibitor E64 or px458-253r. B cells alone and DC cells alone had slight difference among different treatment. **(B)** Concentration of IFN-γ: when CTSL was overexpressed or increased in co-cultured cells, the concentration increased; by contrast, the concentration decreased when CTSL was inhibited or knockdown; single cells had a slight change. **(C, D)** Concentration of IL-6 and IL-10: neither IL-6 nor IL-10 showed significant differences among the three cell types. Data are representative of three independent experiments (*p<0.05, **p<0.01, ***p<0.001).

TGF-β and IFN-γ had been up-regulated significantly when CTSL accumulated in DCs+B cells which infected with *M*.*hp*. On the contrary, the two cytokines had been down-regulated by inhibition of CTSL with E64 or knockdown of CTSL (Fig 3A and 3B). These results suggest that CTSL could induce higher level of TGF-β which functions on promoting B cells switching from IgM to IgA and IFN-γ which plays an important role during *M*.*hp* infection. In addition, the level of TGF-β and IFN-γ did not change significantly from each other in the DC cells or B cells groups (Fig 3A and 3B). However, neither IL-6 nor IL-10 had significant influence along with CTSL (Fig 3C and 3D).

### Cathepsins involved in the processing of Ii chain *in vitro*

**Fig 4:**
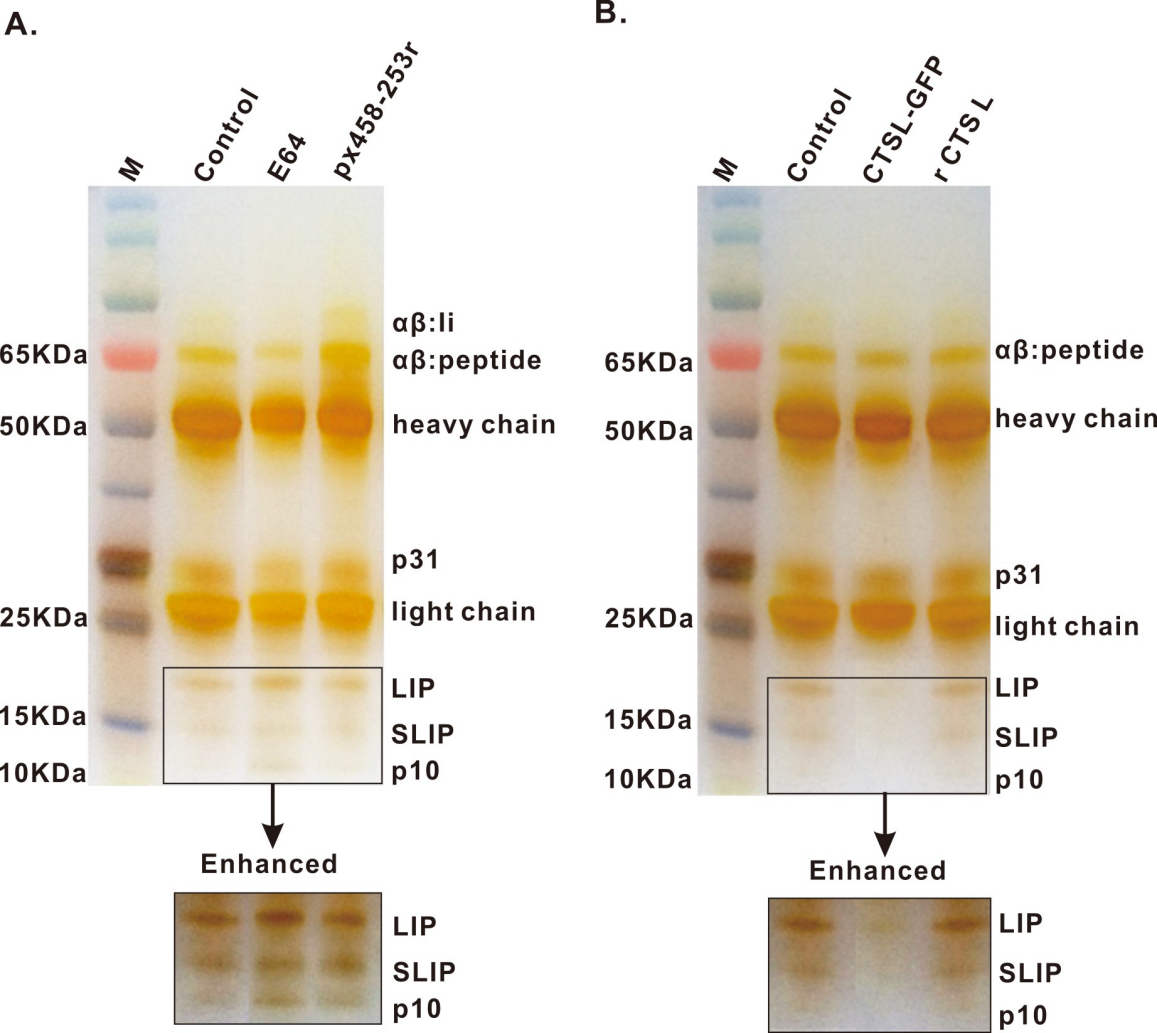
Ii intermediates were detected in DCs+B cells by IP. **(A)** Ii intermediates, LIP, SLIP and p10 accumulated when cells were treated with 10 μg E64 or transinfected with 2 μg px458-253 vector first and incubated with *M*.*hp* later. **(B)** less LIP or SLIP and no p10 accumulated in cells when cells overexpressed with 2 μg CTSL-GFP or add 30 μg rCTSL *in vitro*. Data are representative of three independent experiments.

To test the role of CTSL in the processing of the Ii chain *in vitro*, we analyzed the kinetics of Ii degradation and the accumulation of Ii intermediates in cell models under different conditions. In DCs+B cells, the degradation intermediates of MHC II-associated Ii chain such as Ii like LIP (21~22kDa), SLIP (12~14kDa) and even the smallest fragment p10 accumulated in the group treated with cysteine proteinases inhibitor E64 (Fig 4A). Additionally, when CTSL was overexpressed with eukaryotic plasmid CTSL-GFP, there were no Ii intermediates, neither LIP nor SLIP, were found, and the terminal fragments of Ii were not detected either. But recombinant CTSL protein played no effect on Ii degradation *in vitro* (Fig 4B). Thus, our data suggested that CTSL is involved in the processing of Ii chain by degrading LIP, SLIP and p10 selectively to smaller fragments, and it is the molecular mechanism by which CTSL modulates SIgA response to *M*.*hp* infection.

### SIgA and CTSL were induced coincidently in pigs by challenge with *M*.*hp*

**Fig 5:**
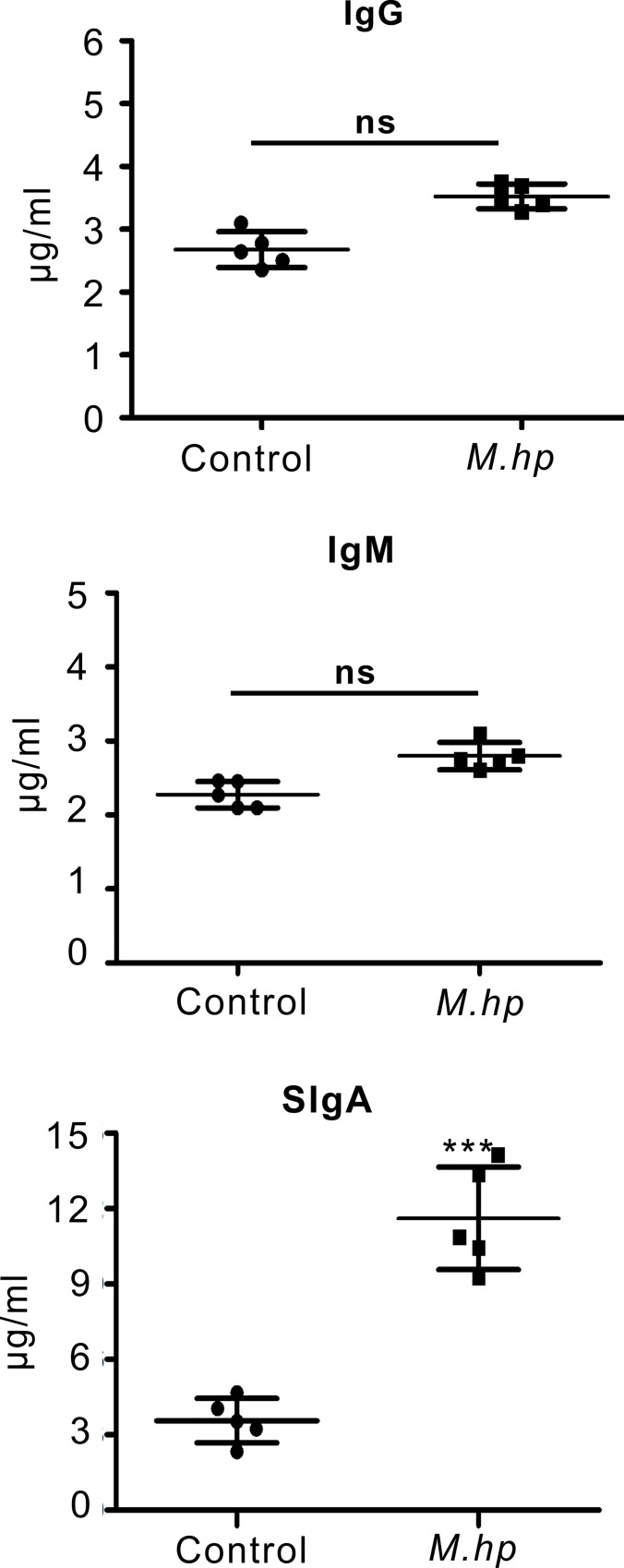
Levels of mycoplasma specific-Ig measured by complete ELISA in BALF samples at 21 DPI. SIgA was significantly higher in *M*.*hp* infection group than control. Data are representative of three independent experiments (***p<0.001).

**Fig 6:**
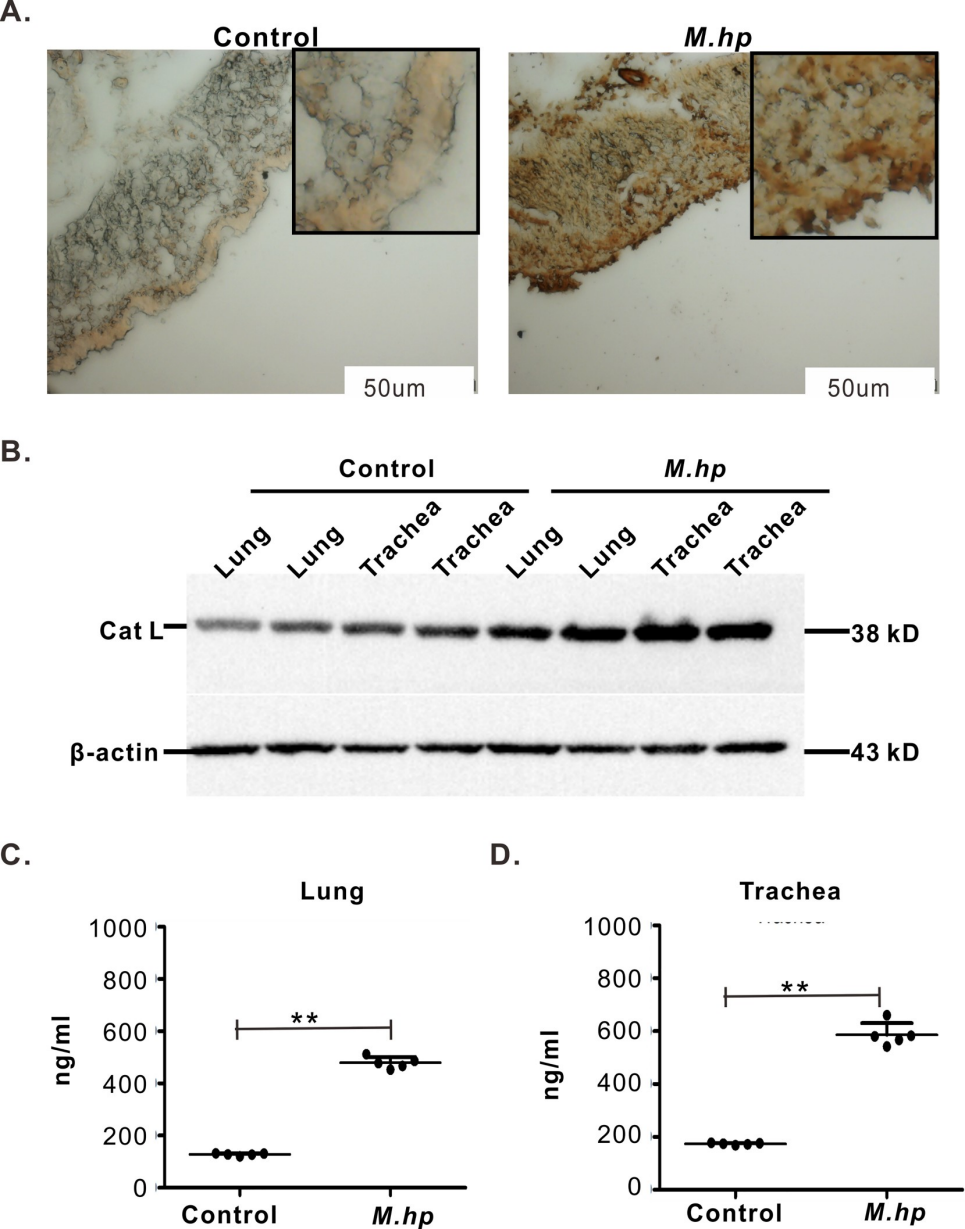
Detection of CTSL. **(A)** Immunohistochemistry of lungs: *M*.*hp* infection group showed positive staining for CTSL, while control group had only weak staining. Positive signals were depicted as a more diffuse area at alveolus. Measure bar = 50 μm. **(B)** CTSL detected by western blot: CTSL were higher in *M*.*hp* infection group (lane 5~8) than in control (lane 1~ 4). **(C, D)** Quantitative analysis of CTSL in trachea and lungs: CTSL concentrations in *M*.*hp* infection group were higher than control. Data are representative of three independent experiments (**p<0.01).

In BALFs of the pigs challenged with *M*.*hp*, SIgA rose over 4 fold from 7 d to 14 d and remained around 15 μg/ml at 21 DPI with a level almost 3 times higher than the control pigs injected with equal volume of sterilized saline water. However, there were no significant differences on the levels of IgG and IgM between the infection group and the control group (Fig 5). In serum, nearly no IgG and IgM were detected and no difference could be seen between the two groups (S3A Fig). In nasal swabs, the concentrations of SIgA and IgM were low but increased by *M*.*hp* infection, while almost no IgG was detected (S3B Fig). The different responses of these three immunoglobulins to *M*.*hp* infection suggested that mucosal immunity (SIgA) is the main immune response pattern of the pigs to *M*.*hp* infection. CTSL was widespread in the tracheas and lungs of the healthy pigs, but increased markedly in the alveolar septum, alveoli, tracheas and lungs of the pigs challenged with *M*.*hp* (Fig 6A). CTSL were overexpressed in lung and trachea samples when pigs infected with *M*.*hp* (Fig 6B). As determined by sandwich ELISA, the concentrations of CTSL in lungs and tracheas rose nearly 3 fold at 21 DPI (Fig 6C and 6D), which coincide with the change in SIgA. Therefore, CTSL expression was correlated with *M*.*hp* infection.

### Recombinant CTSL provided considerable protections against *M*.*hp* infection

**Fig 7:**
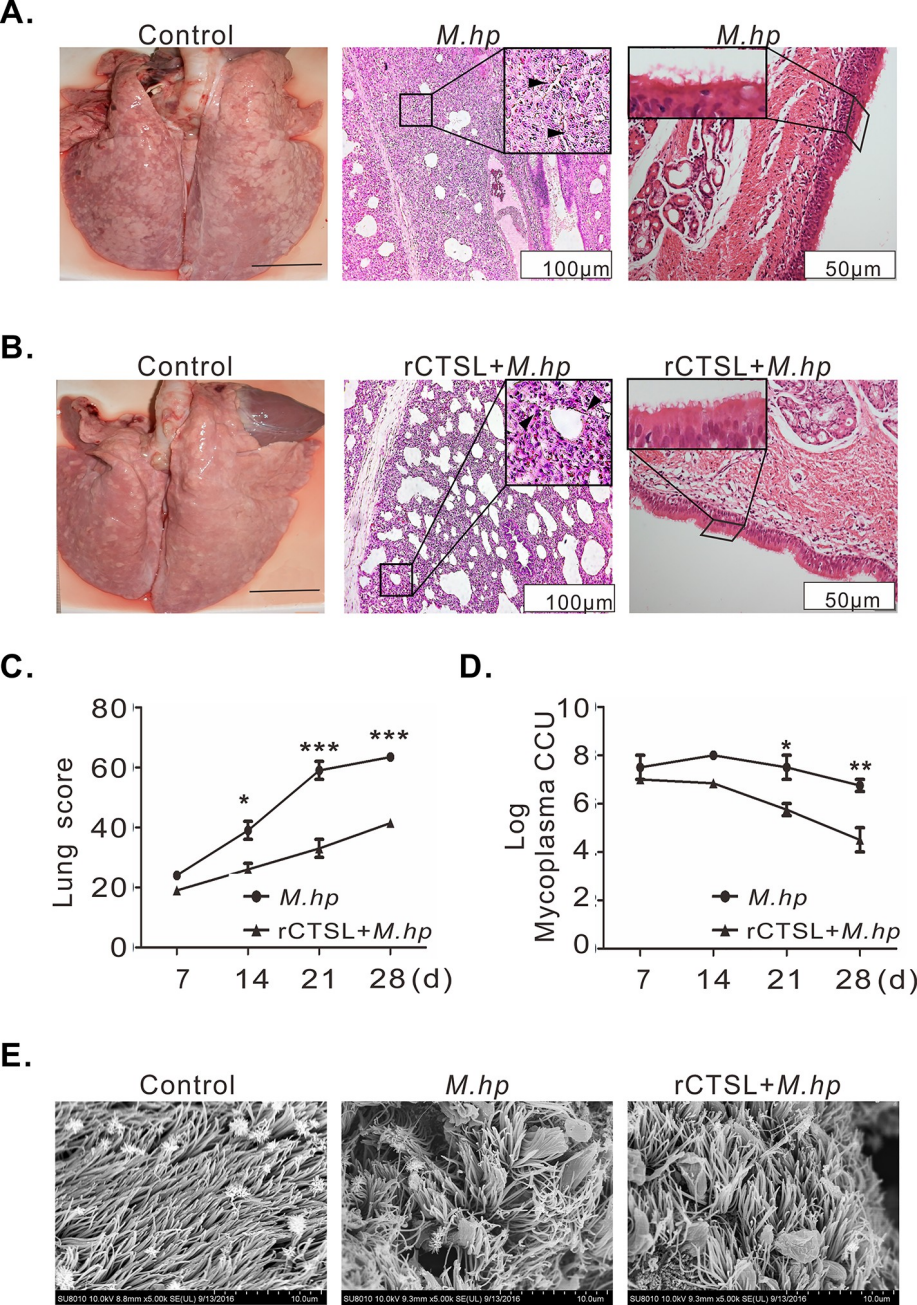
CTSL protected pigs from *M*.*hp* infection. **(A)** *M*.*hp* group: there were multi lobular consolidation of lungs (left); HE staining showed alveolar septa thickened (arrow) and bronchioles were infiltrated with multiple inflammatory cells (arrow) (middle); cilia falling off and disorderly arranged (right). **(B)** rCTSL+*M*.*hp* group: only diaphragmatic lobular showed lesions (left); there was less inflammation around bronchioles and the alveolar septa was moderately thickened (arrow) (middle); cilia partially lost (left). Measure bar = 3cm (left),100 μm (middle), 50 μm (right). **(C)** Lung scores: scores were significant higher at 21 and 28 DPI in *M*.*hp* group than in rCTSL+*M*.*hp* group. **(D)** Live mycoplasma in lungs was lower in rCTSL+*M*.*hp* group than in *M*.*hp* group. **(E)** SEM images of tracheal: cilia appeared sparse and aggregated after *M*.*hp* infection; while arranged almost orderly in rCTSL+*M*.*hp* group. **(A, B, E)** samples from 21 DPI **(C, D)** Data represent means ± SEM from three independent experiments (*p<0.05, **p<0.01, ***p<0.001).

To verify the resistant effect of CTSL on *M*.*hp* infection, rCTSL was used to treat the pigs before they were challenged with *M*.*hp*. Piglets in *M*.*hp* infection group developed typical symptoms such as coughing, running nose, wheezing and fever (S4A Fig). Meanwhile the macroscopic lesions of lungs were serious at 21 DPI, and alveolar septa thickened with macrophage and other inflammatory cells infiltration, and bronchioles/terminal bronchioles hyperplasia were micropathologically visible and most of the tracheal cilia were lost (Fig 7A). On the contrary, piglets treated with rCTSL before challenge showed slight symptoms (S4B Fig). And the histopathological damage of lungs was mild with slight thicker alveolar septa and fewer cilia lost at 21 DPI (Fig 7B). Moreover, the lung lesion scores were lower than *M*.*hp* infection and reduced almost by half at 21 and 28 DPI (Fig 7C), the titer of live mycoplasma also decreased to 10^5.75^ and 10^4.5^ at 21 and 28 DPI separately after treated with rCTSL (Fig 7D). Normal cilia arranged in parallel and appeared to be similar in length and diameter, but clumped after infection and there were mycoplasma-like particles adhering to the ciliated epithelial cells, though meanwhile the cilia was not damaged in the rCTSL+*M*.*hp* group (Fig 7E). These data suggested that rCTSL could provide effective protections for piglets against pneumonia caused by *M*.*hp* infection.

### Recombinant CTSL enhanced the immune responses in pigs

**Fig 8:**
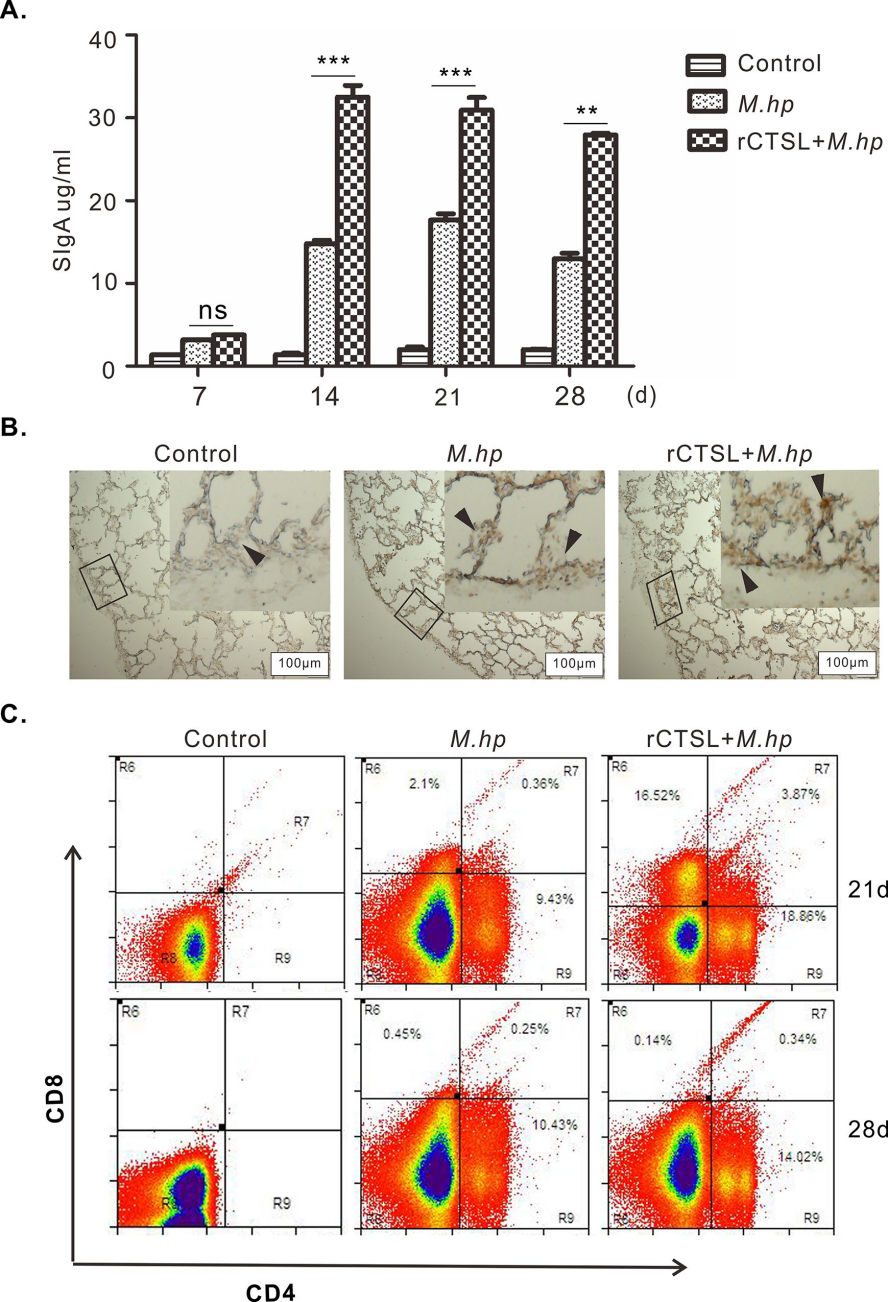
rCTSL enhanced immune responses. **(A)** SIgA in BALFs: the level of SIgA significantly increased at 14 and 21 DPI when treated with rCTSL, and higher than *M*.*hp* group. Data represent means ± SEM from three independent experiments (**p<0.01, ***p<0.001). **(B)** MHC class II molecules were detected by IHC in lung samples: there was positive staining in lungs when pigs were infected with *M*.*hp* (arrow); reaction was stronger in rCTSL+*M*.*hp* group (arrow). **(C)** FACS analysis of CD4 and CD8: CD8^+^T cells abounded in rCTSL+*M*.*hp* group at 21 DPI, CD4^+^ T cells stayed along with infection especially at the late stage.

The concentrations of SIgA in BALFs rose over 4 fold at each time point after challenged with *M*.*hp*, and they almost doubled by treatments with rCTSL in addition to challenge (Fig 8A). As one key molecular on initiating immune response, MHC II was also detected in alveolar septa of pigs and increased obviously after challenged with *M*.*hp* comparing to the control group, with relatively higher rise achieved by rCTSL (Fig 8B). Accordingly, the population of CD4^+^ T lymphocytes widespread in pigs at 21 and 28 DPI with *M*.*hp*, and relatively higher percentages of CD4^+^ T cells were obtained by rCTSL treatment before challenge. There were no CD8^+^ T lymphocytes observed at 21 and 28 DPI in the infection group, whereas CD8^+^ T lymphocytes were existed in the group treated with rCTSL at 21 DPI (Fig 8C). These data suggested that rCTSL enhanced the mucosal immune response to *M*.*hp* infection at least by up-regulating MHCII, which activated CD4^+^ T cells potentially by binding and presenting antigens.

## Discussion

It has been proved that the antigen-specific SIgA induced by pathogens or vaccines, constitutes a first-line defense and confers to effective protections against mucosal infectious diseases, primarily by immune exclusion of viral or bacterial pathogens or neutralizing microbial toxins and pathogens [43–45]. In this study, the levels of SIgA in pig BALFs increased significantly at 21 DPI (Fig 5) while almost no IgG and IgM were detected in sera (S3A Fig), indicating mucosal immune was the main immune responses to *Mycoplasma* infections as reported previously [46, 47]. In airways, antigen-specific SIgA is released by epithelial cells after the association of secretory component (SC) with polymeric IgA, which is produced by IgA-secreting plasma cells differentiated from IgA+ effector B cells. The latter originates from the B cells undergone IgA class-switch recombination (CSR) in response to CD40 ligand (CD40L) and transforming growth factor-β1 (TGFβ1) from activated T cells. Interleukin-5 (IL-5), IL-6 and IL-10, as well as B-cell-activating factor (BAFF) and a proliferation-inducing ligand (APRIL) from DCs are required for the expression of IgA, and the differentiation could be enhanced by APRIL secreted by epithelial cells and DCs [48]. *In vitro* studies with human and mouse cells have shown that the interaction between B cells and DCs is required to achieve a successful IgA class switch, DCs play a critical role in B cell development and B lymphocytes have an important regulatory effect on the Ag-presenting function of DCs *in vitro* and *in vivo* [10, 49, 50]. CTSL is widespread in macrophages derived from different tissues with cathepsin S proteins in their active forms, but its immune related effect in macrophages was not as striking as in DCs and B cells. In this study, stronger responses of CTSL were present in DCs rather than macrophages after challenged with *M*.*hp*, implying DCs+B cells coculturing is a preferred model to study the correlation between CTSL and IgA responses of pigs *in vitro* when transgenic pigs are unavailable. In this report, SIgA response was induced obviously in the cocultured B cells-DCs after infected with *M*.*hp*, and SIgA levels varied in consistent with the changes of CTSL activity by knockdown with CRISPR/Cas9 plasmid, overexpression with CTSL-GFP or inhibited with E64, although E64 could react with some other cysteine proteases such as Cat B, Cat H and Cat S (Fig 2B and 2D). These results revealed that CTSL play a major part in promoting the SIgA response of cocultured DCs +B cells to *M*.*hp* infection.

CTSL is particularly interesting, as it is widely distributed in a variety of tissues and plays critical roles in a variety of biological processes, especially in immune responses and antigen processing [12, 51, 52]. Among endopeptidase cysteine proteases, cathepsin B, F, H, K, L, S and W belong to cathepsin L-like family because of their sequence alignment and traditional functional classification. Phylogeny construction demonstrates that CTSL, Cat S, and Cat K evolved from a common ancestral gene and located in same gene clades. However, the identity between CTSL and Cat S, Cat K was only 56.4% and 58.7% respectively in *Sus scrofa*. Sequence alignments show that mouse CTSL, human CTSL, and human Cat V have about 75% amino acid identities, while CTSL of *Sus scrofa* shared higher homologous identity 84.2% with human and 78.2% with mouse. These alignment data suggest that pig is an ideal animal model to study the roles of CTSL in immune responses to mycoplasma infection instead of mice. In this study, CTSL was found widespread in tracheas and lungs, and raised nearly 3 fold at 21 DPI (Fig 6). In the BALFs of the infected pigs, elevated SIgA was detected early at 7 DPI, and almost 2 or 3 fold higher than the control pigs at 14 and 21 DPI (Fig 8A), demonstrating that there was obvious and positive correlation between CTSL levels and SIgA responses.

To investigate the mechanism of the action of CTSL in inducing SIgA responses, the degradation of Ii chain were determined in DCs+B cells coculturing model after different treatments. We found accumulations of LIP, SLIP and p10 in CTSL-deficient DCs+B cells (Fig 4), suggesting that CTSL was involved in the processing of Ii chain by degrading LIP, SLIP and p10 selectively to smaller fragments, by which CTSL modulates SIgA response to *M*.*hp* infection. Similar effects on Ii chain degradation was found on mice CTSL in cortical thymic epithelium which are responsible for positive selection of CD4^+^ cells and mice Cat S in B cells and DCs as reported before, suggesting CTSL and Cat S play an indispensable role in MHC class II-mediated antigen presentation by degrading Ii intermediates and rendering peptide loading, whereas other cysteine cathepsins appear to be redundant [53–55] [16, 17, 19]. However, CTSL and Cat V/L2 were shown not to participate in Ii-p10 processing in human epithelial cell line HeLa-CIITA and MelJuSo systems expressing Ii chain and HLA-DR, and Cat S is the only cysteine protease able to efficiently process the human p10 fragment *in vivo* [56]. Meanwhile, the use of E64 resulted in critical inhibition in Ii degradation with more accumulation of LIP and SLIP fragments, since E64 could inhibit many cysteine proteases besides CTSL, such as Cat B, H, S, which may mediate the late stages of Ii degradation as well. Surprisingly, no accumulation of LIP and SLIP was found but MHC class II peptide loading was enhanced when CTSL was overexpressed or recombinant CTSL was added. We also did not find the terminal key residue CLIP (~3 kDa) because CLIP had been liberated from the peptide-binding groove by SLA-DM during detection. Although a precise picture of mouse Ii chain degradation has emerged from these observations, little information is available on pig and human Ii chain. The existence of a close homologue of CTSL, the pig Cat V is also sufficiently intriguing to investigate the conditions of pig and human Ii chain degradation.

CTSL is able to broadly affect the immune system by various mechanisms in addition to degrading Ii Chain. It has been found that a mutation in the CTSL gene influences the levels of ECM components in lymphoid organs, the thymic output, and the number of T cells in the periphery. Deficiency of CTSL contributed to blunted cellular immune responses of mice infected with *M*. *pulmonitis* or *orthomyxovirus influenza A* infection [23], which is also confirmed by inhibition of CTSL with downregulated Th1 response and/or upregulated Th2 response in both BALB/c and DBA/2 mice after infection [57] [28]. Besides, CTSL facilitated apoptosis in macrophages and neutrophils and TLR9-mediated signaling although their exact effects on immunity remain undefined [58, 59]. The results of the experiments presented showed that CTSL activity is increased in alveolar septum, alveoli, tracheas and lungs in association with the inflammatory response after infection, as has been found in mice infected with *Pseudomonas aeruginosa* [60]. At the same time, the population of CD4^+^ T cell increased and this differentiation tendency of T lymphocytes was speeded up together with increased MHC II in alveolar septa by injecting rCTSL (Fig 8B and 8C).

Consistent with its effect on immune responses to *M*.*hp* infection, rCTSL provided considerable defense against infection as manifested by reduced lung lesion scores and bacteria burden (Fig 7C and 7D). It has been confirmed that CTSL helps contain pathogenic infections as reflected with higher mortality and lung burdens of *Mycoplasma pulmonitis* or *orthomyxovirus influenza A* in CTSL^-/-^ mice and exacerbated leishmaniasis in mice treated with specific inhibitor of CTSL, while the dissemination and colonization of *Edwardsiella tarda* was suppressed in Japanese flounder with overexpressed CTSL [18, 28, 61]. However, unlike its effect on the above pathogens including influenza virus, CTSL seem to augment *Ebola*, *Hendra*, and severe acute respiratory syndrome viruses to enter cells, thereby advancing these infections by cleaving viral proteins [23, 62, 63]. Interestingly, *Mycobacterium tuberculosis* induced a general down-regulation of cathepsin expression in infected cells, and inhibited IFN γ-mediated mature and increase of CTSL, which favored bacterial survival within human primary macrophage [64].

## Conclusion

In conclusion, CTSL plays crucial roles in innate and adaptive immune response and may bring about different and even opposite effects on the infections depending on the pathogens. Our results suggested that CTSL provide obvious protection against *M*.*hp* for pigs through promoting mucosal immune with definite actions on facilitating MHC class II peptide presentation and CD4^+^T lymphocyte differentiation, although further studies should be undertaken to clarify the exact mechanisms considering the complexity of mucosal immune system and CTSL involved in many pathologic process.

## Supporting information

S1 Fig Microscopic observation of DCs in its growth period. (TIF)

S2 Fig Microscopic observation of B cells in its growth period. (TIF)

S3 Fig Levels of mycoplasma-specific Ig measured by complete ELISA in serum and nasal samples at 21 DPI. (TIF)

S4 Fig Clinical symptoms of piglets when *M*.*hp* infection. (TIF)

S1 Methods CTSL sequence. (DOCX)

S2 Methods Sandwich ELISA construction. (DOCX)

S3 Methods Peripheral blood mononuclear cells (PBMC) isolation. (DOCX)

S4 Methods Flow cytometry analysis (FCM). (DOCX)

S5 Methods Plasmid construction. (DOCX)

## References

[1] Brandtzaeg P. Induction of secretory immunity and memory at mucosal surfaces. Vaccine. 2007;25(30):5467–84. 10.1016/j.vaccine.2006.12.001 17227687

[2] Brandtzaeg P. Secretory IgA: Designed for Anti-Microbial Defense. Frontiers in immunology. 2013;4:222 10.3389/fimmu.2013.00222 23964273 PMC3734371

[3] Tlaskalova-Hogenova H, Stepankova R, Hudcovic T, Tuckova L, Cukrowska B, Lodinova-Zadnikova R, et al Commensal bacteria (normal microflora), mucosal immunity and chronic inflammatory and autoimmune diseases. Immunology letters. 2004;93(2–3):97–108. 10.1016/j.imlet.2004.02.005 15158604

[4] Strugnell RA, Wijburg OL. The role of secretory antibodies in infection immunity. Nature reviews Microbiology. 2010;8(9):656–67. 10.1038/nrmicro2384 20694027

[5] Randall TD, Mebius RE. The development and function of mucosal lymphoid tissues: a balancing act with micro-organisms. Mucosal immunology. 2014;7(3):455–66. 10.1038/mi.2014.11 24569801

[6] Parida S, Anderson J, Cox SJ, Barnett PV, Paton DJ. Secretory IgA as an indicator of oro-pharyngeal foot-and-mouth disease virus replication and as a tool for post vaccination surveillance. Vaccine. 2006;24(8):1107–16. 10.1016/j.vaccine.2005.09.006 16203061

[7] Leigh SA, Branton SL, Evans JD, Collier SD. Effect of infection route and concurrent infectious bronchitis virus vaccination on Mycoplasma gallisepticum disease pathology in an experimental model. Avian pathology: journal of the WVPA. 2012;41(5):497–503. 10.1080/03079457.2012.721925 23025670

[8] Abusugra I, Morein B. Iscom is an efficient mucosal delivery system for Mycoplasma mycoides subsp. mycoides (MmmSC) antigens inducing high mucosal and systemic antibody responses. FEMS Immunology & Medical Microbiology. 1999;23(1):5–12. 10030541 10.1111/j.1574-695X.1999.tb01710.x

[9] Feng Z-x, Shao G-q, Liu M-j, Wu X-s, Zhou Y-q, Gan Y. Immune Responses to the Attenuated Mycoplasma hyopneumoniae 168 Strain Vaccine by Intrapulmonic Immunization in Piglets. Agricultural Sciences in China. 2010;9(3):423–31.

[10] Reboldi A, Arnon TI, Rodda LB, Atakilit A, Sheppard D, Cyster JG. IgA production requires B cell interaction with subepithelial dendritic cells in Peyer's patches. Science. 2016;352(6287):aaf4822 10.1126/science.aaf4822 27174992 PMC4890166

[11] Sadegh-Nasseri S. A step-by-step overview of the dynamic process of epitope selection by major histocompatibility complex class II for presentation to helper T cells. F1000Research. 2016;5. 10.12688/f1000research.7664.1 PMC4902097 27347387

[12] Hughes CE, Benson RA, Bedaj M, Maffia P. Antigen-Presenting Cells and Antigen Presentation in Tertiary Lymphoid Organs. Frontiers in immunology. 2016;7:481 10.3389/fimmu.2016.00481 27872626 PMC5097899

[13] Sadegh-Nasseri S, Kim A. Exogenous antigens bind MHC class II first, and are processed by cathepsins later. Molecular immunology. 2015;68(2 Pt A):81–4. 10.1016/j.molimm.2015.07.018 26254987 PMC4623955

[14] Ferrante A, Gorski J. A Peptide/MHCII conformer generated in the presence of exchange peptide is substrate for HLA-DM editing. Scientific reports. 2012;2:386 10.1038/srep00386 22545194 PMC3338121

[15] Kim A, Hartman IZ, Poore B, Boronina T, Cole RN, Song N, et al Divergent paths for the selection of immunodominant epitopes from distinct antigenic sources. Nature communications. 2014;5:5369 10.1038/ncomms6369 25413013 PMC4241505

[16] Li Q, Ao J, Mu Y, Yang Z, Li T, Zhang X, et al Cathepsin S, but not cathepsin L, participates in the MHC class II-associated invariant chain processing in large yellow croaker (Larimichthys crocea). Fish & shellfish immunology. 2015;47(2):743–50. 26475363 10.1016/j.fsi.2015.10.009

[17] Hsieh CS, deRoos P, Honey K, Beers C, Rudensky AY. A Role for Cathepsin L and Cathepsin S in Peptide Generation for MHC Class II Presentation. The Journal of Immunology. 2002;168(6):2618–25. 11884425 10.4049/jimmunol.168.6.2618

[18] Onishi K, Li Y, Ishii K, Hisaeda H, Tang L, Duan X, et al Cathepsin L is crucial for a Th1-type immune response during Leishmania major infection. Microbes and infection. 2004;6(5):468–74. 10.1016/j.micinf.2004.01.008 15109961

[19] Weitoft T, Larsson A, Manivel VA, Lysholm J, Knight A, Ronnelid J. Cathepsin S and cathepsin L in serum and synovial fluid in rheumatoid arthritis with and without autoantibodies. Rheumatology. 2015;54(10):1923–8. 10.1093/rheumatology/keu486 26060322

[20] Liang JZ, Rao YZ, Lun ZR, Yang TB. Cathepsin L in the orange-spotted grouper, Epinephelus coioides: molecular cloning and gene expression after a Vibrio anguillarum challenge. Fish physiology and biochemistry. 2012;38(6):1795–806. 10.1007/s10695-012-9676-3 22723013

[21] Villa-Mancera A, Reynoso-Palomar A, Utrera-Quintana F, Carreon-Luna L. Cathepsin L1 mimotopes with adjuvant Quil A induces a Th1/Th2 immune response and confers significant protection against Fasciola hepatica infection in goats. Parasitology research. 2014;113(1):243–50. 10.1007/s00436-013-3650-6 24218177

[22] Arockiaraj J, Gnanam AJ, Muthukrishnan D, Thirumalai MK, Pasupuleti M, Milton J, et al Macrobrachium rosenbergii cathepsin L: molecular characterization and gene expression in response to viral and bacterial infections. Microbiological research. 2013;168(9):569–79. 10.1016/j.micres.2013.04.007 23669240

[23] Xu X, Greenland JR, Gotts JE, Matthay MA, Caughey GH. Cathepsin L Helps to Defend Mice from Infection with Influenza A. PloS one. 2016;11(10):e0164501 10.1371/journal.pone.0164501 27716790 PMC5055332

[24] Wang R, Song L, Su B, Zhao H, Zhang D, Peatman E, et al Mucosal expression signatures of two Cathepsin L in channel catfish (Ictalurus punctatus) following bacterial challenge. Fish & shellfish immunology. 2015;47(1):582–9. 26434716 10.1016/j.fsi.2015.09.047

[25] Lu HJ, Yan J, Jin PY, Zheng GH, Qin SM, Wu DM, et al MicroRNA-152 inhibits tumor cell growth while inducing apoptosis via the transcriptional repression of cathepsin L in gastrointestinal stromal tumor. Cancer biomarkers: section A of Disease markers. 2018;21(3):711–22. 10.3233/CBM-170809 PMC13078309 29278883

[26] Burton LJ, Henderson V, Liburd L, Odero-Marah VA. Snail transcription factor NLS and importin beta1 regulate the subcellular localization of Cathepsin L and Cux1. Biochemical and biophysical research communications. 2017;491(1):59–64. 10.1016/j.bbrc.2017.07.039 28698143 PMC5568889

[27] van der Torren CR, Verrijn Stuart AA, Lee D, Meerding J, van de Velde U, Pipeleers D, et al Serum Cytokines as Biomarkers in Islet Cell Transplantation for Type 1 Diabetes. PloS one. 2016;11(1):e0146649 10.1371/journal.pone.0146649 26751709 PMC4713434

[28] Xu X, Greenland J, Baluk P, Adams A, Bose O, McDonald DM, et al Cathepsin L protects mice from mycoplasmal infection and is essential for airway lymphangiogenesis. American journal of respiratory cell and molecular biology. 2013;49(3):437–44. 10.1165/rcmb.2013-0016OC 23600672 PMC3824055

[29] Reiser J, Adair B, Reinheckel T. Specialized roles for cysteine cathepsins in health and disease. The Journal of clinical investigation. 2010;120(10):3421–31. 10.1172/JCI42918 20921628 PMC2947230

[30] Muller S, Faulhaber A, Sieber C, Pfeifer D, Hochberg T, Gansz M, et al The endolysosomal cysteine cathepsins L and K are involved in macrophage-mediated clearance of Staphylococcus aureus and the concomitant cytokine induction. FASEB journal: official publication of the Federation of American Societies for Experimental Biology. 2014;28(1):162–75. 24036885 10.1096/fj.13-232272

[31] Waxman S, Khabbaz K, Connolly R, Tang J, Dabreo A, Egerhei L, et al Intravascular imaging of atherosclerotic human coronaries in a porcine model: a feasibility study. The international journal of cardiovascular imaging. 2008;24(1):37–44. 10.1007/s10554-007-9227-7 17503218

[32] St Goar FG, Fann JI, Komtebedde J, Foster E, Oz MC, Fogarty TJ, et al Endovascular edge-to-edge mitral valve repair: short-term results in a porcine model. Circulation. 2003;108(16):1990–3. 10.1161/01.CIR.0000096052.78331.CA 14530193

[33] Ran FA, Hsu PD, Wright J, Agarwala V, Scott DA, Zhang F. Genome engineering using the CRISPR-Cas9 system. Nature protocols. 2013;8(11):2281–308. 10.1038/nprot.2013.143 24157548 PMC3969860

[34] Westermann AJ, Forstner KU, Amman F, Barquist L, Chao Y, Schulte LN, et al Dual RNA-seq unveils noncoding RNA functions in host-pathogen interactions. Nature. 2016;529(7587):496–501. 10.1038/nature16547 26789254

[35] Marchioro SB, Fisch A, Gomes CK, Jorge S, Galli V, Haesebrouck F, et al Local and systemic immune responses induced by a recombinant chimeric protein containing Mycoplasma hyopneumoniae antigens fused to the B subunit of Escherichia coli heat-labile enterotoxin LTB. Veterinary microbiology. 2014;173(1–2):166–71. 10.1016/j.vetmic.2014.07.009 25091529

[36] Halbur PG, Paul PS, Frey ML, Landgraf J, Eernisse K, Meng X-J, et al Comparison of the Pathogenicity of Two US Porcine Reproductive and Respiratory Syndrome Virus Isolates with that of the Lelystad Virus. Veterinary Pathology. 1995;32(6):648–60. 10.1177/030098589503200606 8592800

[37] Sibila M, Aragón V, Fraile L, Segalés J. Comparison of four lung scoring systems for the assessment of the pathological outcomes derived from Actinobacillus pleuropneumoniaeexperimental infections. BMC veterinary research. 2014;10(1):165. 25038822 10.1186/1746-6148-10-165 PMC4112831

[38] Meyns T, Van Steelant J, Rolly E, Dewulf J, Haesebrouck F, Maes D. A cross-sectional study of risk factors associated with pulmonary lesions in pigs at slaughter. Veterinary journal. 2011;187(3):388–92. 10.1016/j.tvjl.2009.12.027 20122861

[39] Lee JY, Park HJ, Kim YK, Yu S, Chong YP, Kim SH, et al Cellular profiles of bronchoalveolar lavage fluid and their prognostic significance for non-HIV-infected patients with Pneumocystis jirovecii pneumonia. Journal of clinical microbiology. 2015;53(4):1310–6. 10.1128/JCM.03494-14 25673796 PMC4365245

[40] Yamazaki K, Ogura S, Ishizaka A, Oh-hara T, Nishimura M. Bronchoscopic microsampling method for measuring drug concentration in epithelial lining fluid. American journal of respiratory and critical care medicine. 2003;168(11):1304–7. 10.1164/rccm.200301-111OC 12904323

[41] Zhou L, Yang B, Xu L, Jin H, Ge X, Guo X, et al Efficacy evaluation of three modified-live virus vaccines against a strain of porcine reproductive and respiratory syndrome virus NADC30-like. Veterinary microbiology. 2017;207:108–16. 10.1016/j.vetmic.2017.05.031 28757009

[42] Halbur PG, Paul PS, Frey ML, Landgraf J, Eernisse K, Meng X-J, et al Comparison of the Antigen Distribution of Two US Porcine Reproductive and Respiratory Syndrome Virus Isolates with that of the Lelystad Virus. Veterinary Pathology. 1996;33(2):159–70. 10.1177/030098589603300205 8801709

[43] Tokuhara D, Yuki Y, Nochi T, Kodama T, Mejima M, Kurokawa S, et al Secretory IgA-mediated protection against V. cholerae and heat-labile enterotoxin-producing enterotoxigenic Escherichia coli by rice-based vaccine. Proc Natl Acad Sci USA. 2010;107(19):8794–9. 10.1073/pnas.0914121107 20421480 PMC2889329

[44] Taylor HP, Dimmock NJ. Mechanism of neutralization of influenza virus by secretory IgA is different from that of monomeric IgA or IgG. The Journal of Experimental Medicine. 1985;161(1):198–209. 2981953 10.1084/jem.161.1.198 PMC2187541

[45] Seibert CW, Rahmat S, Krause JC, Eggink D, Albrecht RA, Goff PH, et al Recombinant IgA Is Sufficient To Prevent Influenza Virus Transmission in Guinea Pigs. Journal of virology. 2013;87(14):7793–804. 10.1128/JVI.00979-13 23698296 PMC3700183

[46] Feng ZX, Bai Y, Yao JT, Pharr GT, Wan XF, Xiao SB, et al Use of serological and mucosal immune responses to Mycoplasma hyopneumoniae antigens P97R1, P46 and P36 in the diagnosis of infection. Veterinary journal. 2014;202(1):128–33. 10.1016/j.tvjl.2014.06.019 25066030

[47] Feng ZX, Wei YN, Li GL, Lu XM, Wan XF, Pharr GT, et al Development and validation of an attenuated Mycoplasma hyopneumoniae aerosol vaccine. Veterinary microbiology. 2013;167(3–4):417–24. 10.1016/j.vetmic.2013.08.012 24035264

[48] Cerutti A. The regulation of IgA class switching. Nature reviews Immunology. 2008;8(6):421–34. 10.1038/nri2322 18483500 PMC3062538

[49] Bayry J, Lacroix-Desmazes S, Kazatchkine MD, Hermine O, Tough DF, Kaveri SV. Modulation of Dendritic Cell Maturation and Function by B Lymphocytes. The Journal of Immunology. 2005;175(1):15–20. 15972625 10.4049/jimmunol.175.1.15

[50] Morva A, Lemoine S, Achour A, Pers JO, Youinou P, Jamin C. Maturation and function of human dendritic cells are regulated by B lymphocytes. Blood. 2012;119(1):106–14. 10.1182/blood-2011-06-360768 22067387

[51] Duncan EM, Muratore-Schroeder TL, Cook RG, Garcia BA, Shabanowitz J, Hunt DF, et al Cathepsin L proteolytically processes histone H3 during mouse embryonic stem cell differentiation. Cell. 2008;135(2):284–94. 10.1016/j.cell.2008.09.055 18957203 PMC2579750

[52] Chapman HA. Endosomal proteases in antigen presentation. Current opinion in immunology. 2006;18(1):78–84. 10.1016/j.coi.2005.11.011 16338127

[53] Hsing LC, Rudensky AY. The lysosomal cysteine proteases in MHC class II antigen presentation. Immunological Reviews. 2005;207(1):229–41. 16181340 10.1111/j.0105-2896.2005.00310.x

[54] Nakagawa TY, Rudensky AY. The role of lysosomal proteinases in MHC class Il-mediated antigen processing and presentation. Immunological Reviews. 1999;172(1):121–9. 10631942 10.1111/j.1600-065x.1999.tb01361.x

[55] Nakagawa T, Roth W, Wong P, Nelson A, Farr A, Deussing J, et al Cathepsin L: Critical Role in Ii Degradation and CD4 T Cell Selection in the Thymus. Science. 1998;280(5362):450–3. 9545226 10.1126/science.280.5362.450

[56] Bania J, Gatti E, Lelouard H, David A, Cappello F, Weber E, et al Human cathepsin S, but not cathepsin L, degrades efficiently MHC class II-associated invariant chain in nonprofessional APCs. Proceedings of the National Academy of Sciences. 2003;100(11):6664–9. 10.1073/pnas.1131604100 PMC164504 12748383

[57] Onishi K, Li Y, Ishii K, Hisaeda H, Tang L, Duan X, et al Cathepsin L is crucial for a Th1-type immune response during Leishmania major infection. Microbes and infection. 2004;6(5):468–74. 10.1016/j.micinf.2004.01.008 15109961

[58] Blomgran R, Zheng L, Stendahl O. Cathepsin-cleaved Bid promotes apoptosis in human neutrophils via oxidative stress-induced lysosomal membrane permeabilization. Journal of Leukocyte Biology. 2007;81(5):1213–23. 10.1189/jlb.0506359 17264306

[59] Matsumoto F, Saitoh S-i, Fukui R, Kobayashi T, Tanimura N, Konno K, et al Cathepsins are required for Toll-like receptor 9 responses. Biochemical and biophysical research communications. 2008;367(3):693–9. 10.1016/j.bbrc.2007.12.130 18166152

[60] Dong Z, Katar M, Linebaugh BE, Sloane BF, Berk RS. Expression of cathepsins B, D and L in mouse corneas infected with Pseudomonas aeruginosa. European Journal of Biochemistry. 2001;268(24):6408–16. 11737195 10.1046/j.0014-2956.2001.02607.x

[61] Wang JJ, Sun L. Edwardsiella tarda-regulated proteins in Japanese flounder (Paralichthys olivaceus): Identification and evaluation of antibacterial potentials. Journal of proteomics. 2015;124:1–10. 10.1016/j.jprot.2015.04.011 25896741

[62] Marzi A, Reinheckel T, Feldmann H. Cathepsin B & L Are Not Required for Ebola Virus Replication. PLoS neglected tropical diseases. 2012;6(12):e1923 10.1371/journal.pntd.0001923 23236527 PMC3516577

[63] Kawase M, Shirato K, Matsuyama S, Taguchi F. Protease-Mediated Entry via the Endosome of Human Coronavirus 229E. Journal of virology. 2009;83(2):712–21. 10.1128/JVI.01933-08 18971274 PMC2612384

[64] Pires D, Marques J, Pombo JP, Carmo N, Bettencourt P, Neyrolles O, et al Role of Cathepsins in Mycobacterium tuberculosis Survival in Human Macrophages. Scientific reports. 2016;6:32247 10.1038/srep32247 27572605 PMC5004184

