# Supplementary figures and images for: Cathepsin L promotes secretory IgA response by participating in antigen presentation pathways during Mycoplasma Hyopneumoniae infection
Source: PLoS One. 2019 Apr 15;14(4):e0215408. doi: 10.1371/journal.pone.0215408 (PMC6464228; doi:10.1371/journal.pone.0215408)

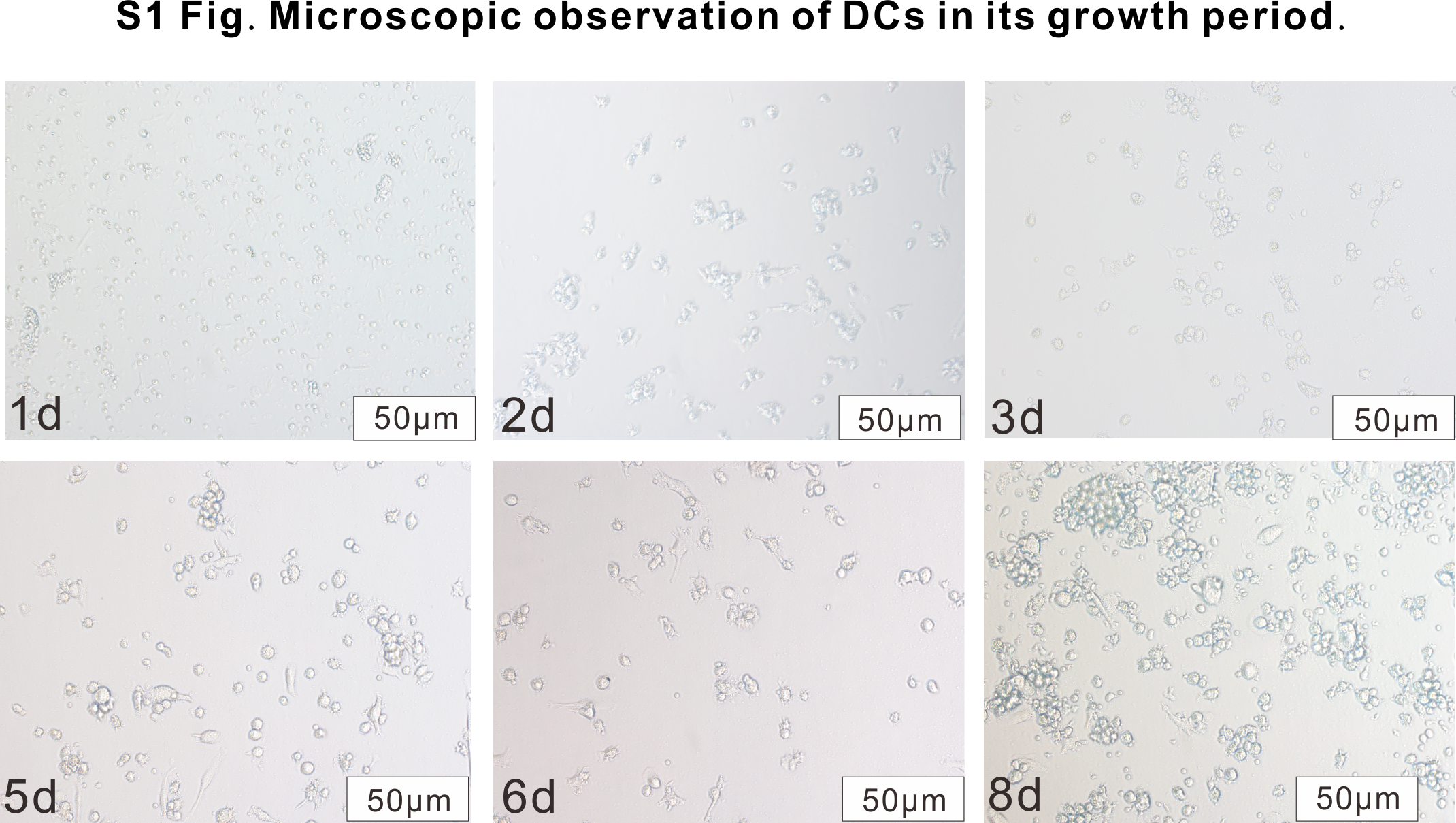

Supplement: S1 Fig — (TIF) [file pone.0215408.s001.tif]

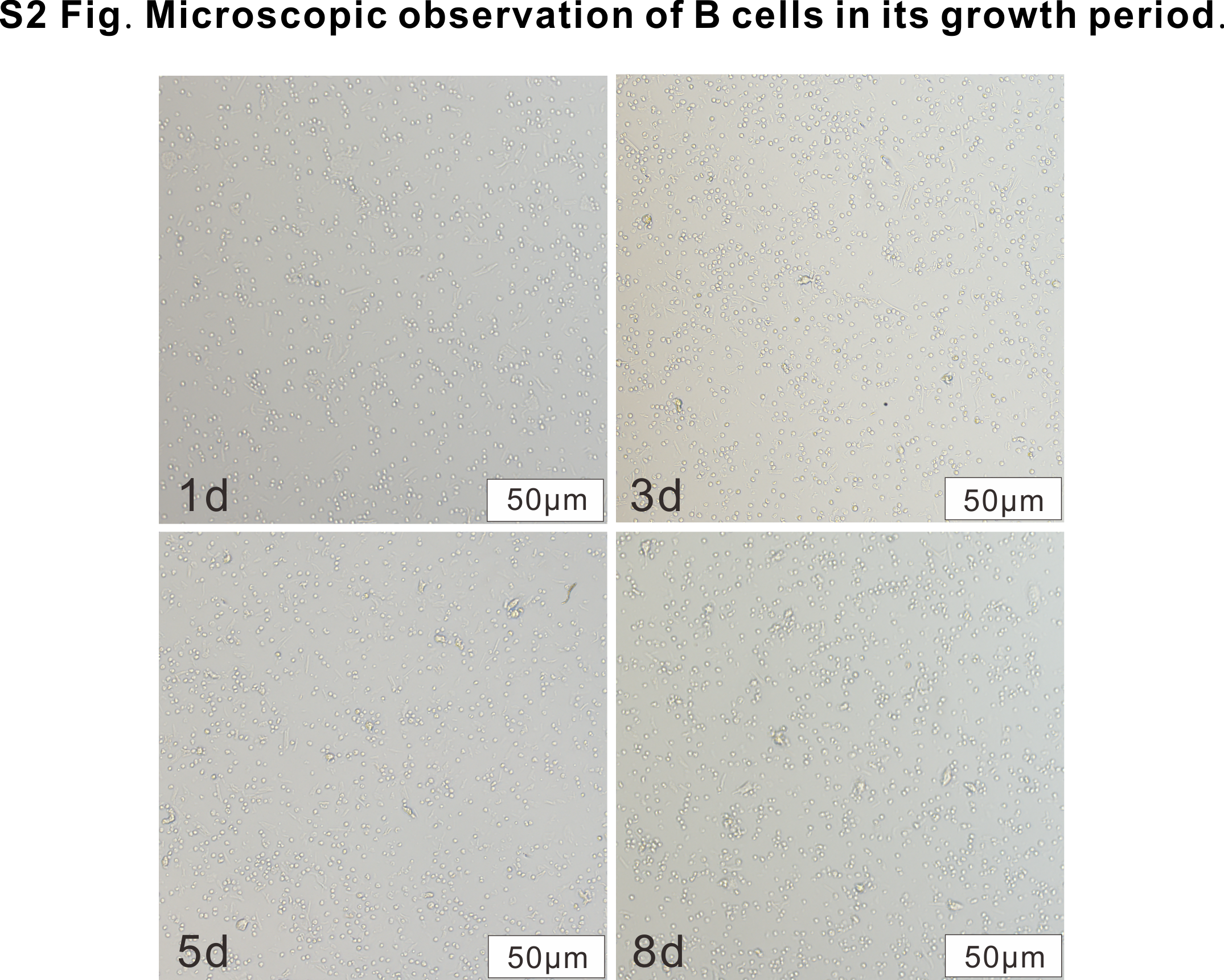

Supplement: S2 Fig — (TIF) [file pone.0215408.s002.tif]

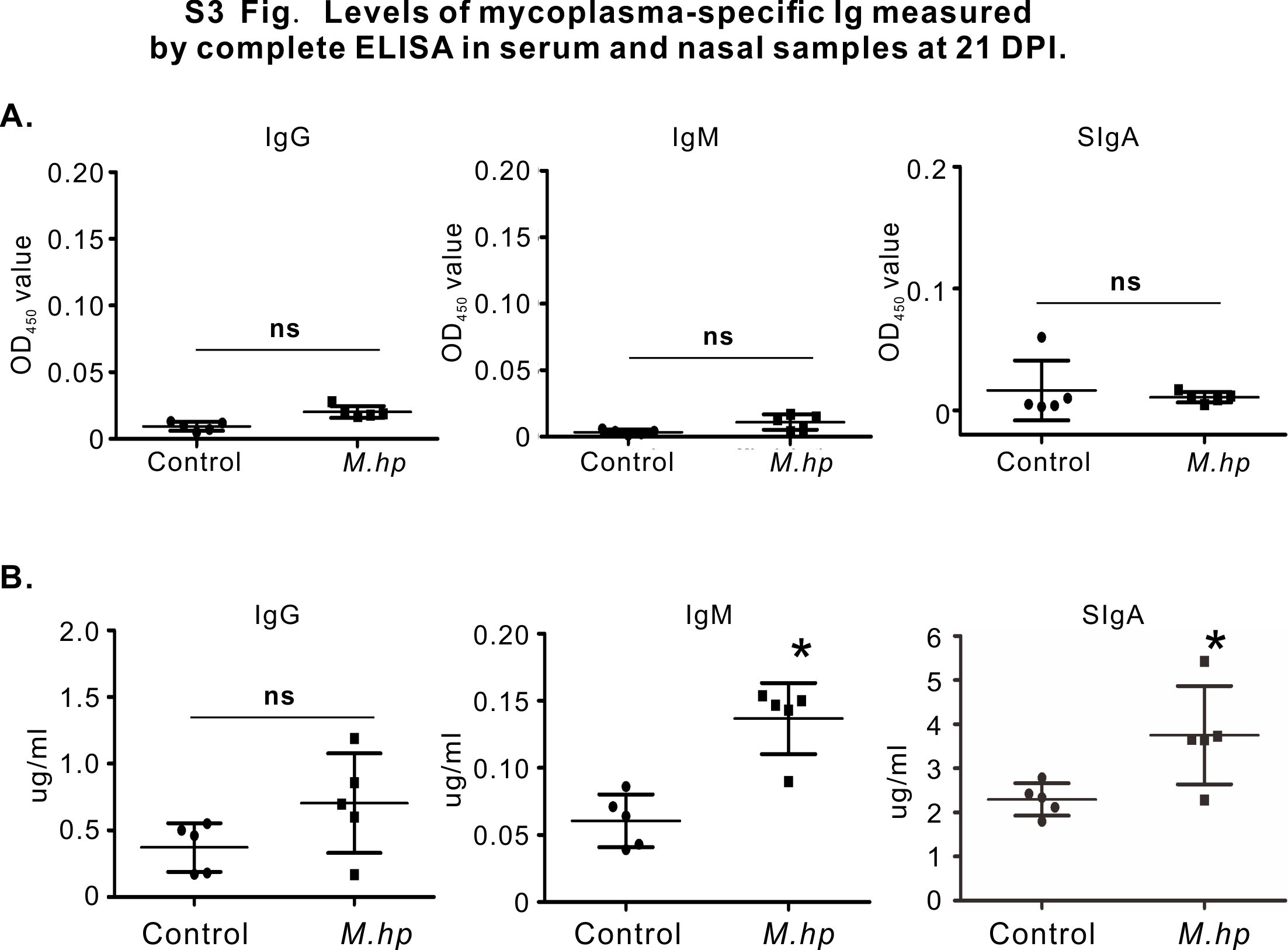

Supplement: S3 Fig — (TIF) [file pone.0215408.s003.tif]
