# Supplementary material for: Cathepsin L promotes secretory IgA response by participating in antigen presentation pathways during Mycoplasma Hyopneumoniae infection
Source: PLoS One. 2019 Apr 15;14(4):e0215408. doi: 10.1371/journal.pone.0215408 (PMC6464228; doi:10.1371/journal.pone.0215408)
Supplement: S1 Methods — (DOCX) [file pone.0215408.s005.docx]

**S1 Methods. CTSL sequence.**

Sus scrofa cathepsin V (CTSV), mRNA, NCBI Reference Sequence: NM_213892.1

>NM_213892.1:78-1082 Sus scrofa cathepsin V (CTSV), mRNA

ATGAAACCTTCACTCTTCCTGACCGCCCTTTGCTTGGGAATAGCCTCAGCTGCTCCAAAACTTGATCAAAATTTAGATGCAGACTGGTACAAGTGGAAGGCAACACACGGGAGACTCTATGGCATGAATGAAGAAGGATGGCGGAGAGCCGTATGGGAGAAGAATATGAAAATGATTGAACTGCACAATCAGGAATACAGCCAAGGGAAACATGGCTTCAGCATGGCCATGAATGCCTTTGGTGACATGACCAATGAAGAATTCAGGCAGGTGATGAATGGCTTTCAAAACCAGAAGCACAAGAAGGGGAAAGTGTTCCACGAATCTCTGGTTCTTGAGGTCCCCAAATCGGTAGATTGGAGAGAAAAAGGCTATGTCACTGCCGTGAAGAATCAGGGTCAGTGTGGTTCTTGTTGGGCTTTTAGTGCCACCGGCGCCCTCGAAGGACAGATGTTCCGGAAAACCGGCAAGCTTGTTTCACTGAGTGAGCAGAACCTGGTGGACTGTTCTCGGCCTCAAGGCAATCAGGGCTGCAATGGTGGCCTAATGGATAATGCCTTCCAGTACGTGAAGGACAATGGAGGCCTGGACACAGAGGAATCCTATCCGTACCTTGGAAGGGAAACAAACAGCTGTACCTATAAGCCCGAGTGTTCTGCTGCCAACGACACCGGCTTCGTGGACATCCCTCAGCGGGAGAAGGCCCTTATGAAGGCAGTCGCAACTGTGGGGCCCATCTCTGTTGCTATTGATGCGGGCCATTCATCTTTCCAGTTCTATAAGTCAGGCATTTATTATGATCCAGACTGCAGCAGCAAAGACCTGGATCATGGTGTTTTGGTGGTTGGCTATGGCTTTGAAGGAACTGATTCAAATAGCAGTAAATTTTGGATTGTCAAGAACAGTTGGGGTCCAGAATGGGGCTGGAACGGCTACGTAAAAATGGCCAAAGACCAGAACAACCACTGTGGAATTTCCACAGCAGCCAGCTATCCCACCGTGTGA
