# Supplementary material for: Cathepsin L promotes secretory IgA response by participating in antigen presentation pathways during Mycoplasma Hyopneumoniae infection
Source: PLoS One. 2019 Apr 15;14(4):e0215408. doi: 10.1371/journal.pone.0215408 (PMC6464228; doi:10.1371/journal.pone.0215408)
Supplement: S2 Methods — (DOCX) [file pone.0215408.s006.docx]

**S2 Methods. Sandwich ELISA construction.**

Briefly, plates were coated with pAb diluted 200-fold with coating buffer (0.05M Na_2_CO_3_-NaHCO_3_, pH 9.6), incubated overnight at 4°C. After that, plates were blocked with 5% skim milk in PBST (PBS containing 0.5‰ Tween-20) for 2 h. Plates were washed with PBST three times, and then 100 μl of standard or samples were added in each well and incubated for 1.5 h at 37°C. After washing, 100 μl of diluted mAb (1:200) were added, and the plates were then incubated at 37°C for 2 h. This was followed by the addition of 100 μl diluted goat anti mouse IgG-HRP (1:5000) and subsequent incubation at 37°C for 0.5 h. Afterwards, plates were washed three times and 100 μl TMB substrate solution (Solarbio, China) were added to each well. After 10 min incubation in the dark, 50 μl stop solution (2M H_2_SO_4_) was added and the absorbance was measured using a microplate reader at 450nm. Data were calculated using a standard curve.
