# Supplementary material for: Cathepsin L promotes secretory IgA response by participating in antigen presentation pathways during Mycoplasma Hyopneumoniae infection
Source: PLoS One. 2019 Apr 15;14(4):e0215408. doi: 10.1371/journal.pone.0215408 (PMC6464228; doi:10.1371/journal.pone.0215408)
Supplement: S3 Methods — (DOCX) [file pone.0215408.s007.docx]

**S3 Methods. Peripheral blood mononuclear cells (PBMC) isolation.**

PBMC were immediately isolated by density gradient centrifugation using pig lymphocyte separation medium (1.110±0.01g/mL) (DAKEWE, DKW33-P0100) after venous extraction and lysis of red blood cells (RBC) with ammonium chloride solution (STEMCELL, Catalog #07800). PBMC were incubated in plates at a concentration of 10^6^ cells/ml in 1640 RPMI medium (GIBCO, USA) with 10% fetal bovine serum (FBS) (GIBCO, USA). After 18h, PBMC were separated from monocytes that had adhered to the plate bottom by washing with phosphate-buffered saline (0.01 M PBS, pH 7.2).
