# Supplementary material for: Cathepsin L promotes secretory IgA response by participating in antigen presentation pathways during Mycoplasma Hyopneumoniae infection
Source: PLoS One. 2019 Apr 15;14(4):e0215408. doi: 10.1371/journal.pone.0215408 (PMC6464228; doi:10.1371/journal.pone.0215408)
Supplement: S4 Methods — (DOCX) [file pone.0215408.s008.docx]

**S4 Methods. Flow cytometry analysis (FCM).**

Cells were collected on days 3 and 8 of culture, and then washed with FCM buffer (calcium and magnesium free PBS pH 7.4, 2% FBS). Viable cells were counted after staining with 0.4% Trypan blue and resuspended in FCM buffer at a concentration of 10^6^ cells/ml. Cells (100 µl) were incubated at 4 ℃ with primary antibodies for 30 minutes. Cells were washed twice with 1ml FCM buffer by centrifugation at 250g for 5 min and incubated with goat anti-mouse IgG conjugated with APC (Allophycocyanin) at a dilution of 1:200 for 30 min in the dark. Cells were then washed twice with 1ml FCM buffer by centrifugation. Finally, cells were resuspended in the FCM buffer, and the data were acquired and analyzed with a FACS flow cytometer (Beckman Coulter). Dead cells were excluded from the analysis by DAPI staining.

Heparin-treated blood samples were collected at DPI 21 and 28. Blood samples were labelled with mouse monoclonal anti-porcine CD4-FITC [clone 74-12-4] and mouse monoclonal anti-pig CD8- PE/Cy5^®^ [clone 76-11-2]. To remove contaminating erythrocytes, a lysing solution was employed (Stem cell, USA). Cell suspensions were analyzed using the flow cytometer. Before acquisition, the optical path was adjusted by testing with the optic calibrator Flow Check (Beckman Coulter). Data acquisition and analysis was performed with the Elite workstation and 10,000 events were acquired per tube. The three major cell populations (i.e., granulocytes, lymphocytes and monocytes) were identified by means of proper gating and compensation in a forward versus side scatter dot plot and then by using fluorescence signals. To determine the absolute numbers of cell populations (i.e., number of cells/µl of blood), the internal standard fluorescence beads (Flow-Count, Beckman Coulter) were used.
