# Supplementary material for: Cathepsin L promotes secretory IgA response by participating in antigen presentation pathways during Mycoplasma Hyopneumoniae infection
Source: PLoS One. 2019 Apr 15;14(4):e0215408. doi: 10.1371/journal.pone.0215408 (PMC6464228; doi:10.1371/journal.pone.0215408)
Supplement: S5 Methods — (DOCX) [file pone.0215408.s009.docx]

**S5 Methods. Plasmid construction.**

The plasmid pET28a-CTSL was digested by double restriction enzyme to recover the target gene fragments. Eukaryotic expression vector pEGFP-N1 was applied with the same digestion. The enzyme reaction contained 2 µl target gene fragment (or vector pEGFP-N1), 2 µl 10 × CutSmart Buffer (NEB), 1µl *Pst*I, 1µl *Kpn*I and ddH_2_O up to 20µl. Under the guidance of the T4 DNA ligase (NEB) system instructions, the purified target fragment of the CTSL gene was directionally ligated into pEGFP-N1 vector in a 10µl reaction system containing 3µl target fragment, 1µl pEGFP-N1,1µl T4 DNA ligase, 2µl Ligation buffer and ddH_2_O up to 10µl. The reactants were at 16℃ overnight and then the ligation was transformed into competent E.*coli* DH5α cells. The target plasmids named CTSL-GFP were extracted from the bacterial liquid according to the instructions of PureYieldTM Plasmid Midiprep System (Promega).
